# Characterization of the human myelin oligodendrocyte glycoprotein antibody response in demyelination

**DOI:** 10.1186/s40478-019-0786-3

**Published:** 2019-09-03

**Authors:** Fiona Tea, Joseph A. Lopez, Sudarshini Ramanathan, Vera Merheb, Fiona X. Z. Lee, Alicia Zou, Deepti Pilli, Ellis Patrick, Anneke van der Walt, Mastura Monif, Esther M. Tantsis, Eppie M. Yiu, Steve Vucic, Andrew P. D. Henderson, Anthony Fok, Clare L. Fraser, Jeanette Lechner-Scott, Stephen W. Reddel, Simon Broadley, Michael H. Barnett, David A. Brown, Jan D. Lunemann, Russell C. Dale, Fabienne Brilot, the Australasian and New Zealand MOG Study Group, Adriane Sinclair, Allan G. Kermode, Andrew Kornberg, Annie Bye, Benjamin McGettigan, Benjamin Trewin, Bruce Brew, Bruce Taylor, Chris Bundell, Christina Miteff, Christopher Troedson, Clair Pridmore, Claire Spooner, Con Yiannikas, Cullen O’Gorman, Damian Clark, Dan Suan, Dean Jones, Dean Kilfoyle, Deepak Gill, Denis Wakefield, Dirk Hofmann, Emily Mathey, Gina O’Grady, Hannah F. Jones, Heidi Beadnall, Helmut Butzkueven, Himanshu Joshi, Ian Andrews, Ian Sutton, Jennifer MacIntyre, Jennifer M. Sandbach, Jeremy Freeman, John King, John H. O’Neill, John Parratt, Joshua Barton, Justin Garber, Kate Ahmad, Kate Riney, Katherine Buzzard, Kavitha Kothur, Laurence C. Cantrill, Manoj P. Menezes, Mark A. Paine, Mark Marriot, Mahtab Ghadiri, Michael Boggild, Mitchell Lawlor, Monica Badve, Monique Ryan, Muhammed Aaqib, Neil Shuey, Nerissa Jordan, Nicholas Urriola, Nicholas Lawn, Owen White, Pamela McCombe, Rakesh Patel, Richard Leventer, Richard Webster, Robert Smith, Sachin Gupta, Shekeeb S. Mohammad, Sekhar Pillai, Simon Hawke, Sumu Simon, Sophie Calvert, Stefan Blum, Stephen Malone, Suzanne Hodgkinson, Tina K. Nguyen, Todd A. Hardy, Tomas Kalincik, Tyson Ware, Victor S. C. Fung, William Huynh

**Affiliations:** 1 Brain Autoimmunity Group, Kids Neuroscience Centre, Kids Research at the Children’s Hospital at Westmead, Locked Bag 4001, Sydney, NSW 2145 Australia; 2 Discipline of Child and Adolescent Health, Faculty of Medicine and Health, The University of Sydney, Sydney, Australia; 3 Discipline of Applied Medical Science, The University of Sydney, Sydney, Australia; 4 School of Mathematics and Statistics, The University of Sydney, Sydney, Australia; 5 Department of Neurosciences, Central Clinical School, Monash University, Melbourne, Australia; 6 Department of Neurology, Royal Children’s Hospital and Neurosciences Research, Murdoch Children’s Research Institute, Melbourne, Australia; 7 Department of Paediatrics, The University of Melbourne, Melbourne, Australia; 8 Department of Neurology, Westmead Hospital, Sydney, Australia; 9 Department of Neurology, Monash Health, Melbourne, Australia; 10 Save Sight Institute, Faculty of Medicine and Health, The University of Sydney, Sydney, Australia; 11 Hunter Medical Research Institute, Newcastle, Australia; 12 Faculty of Medicine and Public Health, The University of Newcastle, Newcastle, Australia; 13 Department of Neurology, John Hunter Hospital, Newcastle, Australia; 14 Brain and Mind Centre, The University of Sydney, Sydney, Australia; 15 Department of Neurology, Concord Repatriation General Hospital, Sydney, Australia; 16 Department of Neurology, School of Medicine, Gold Coast University Hospital, Griffith University, Gold Coast, Australia; 17 New South Wales Health Pathology, Institute of Clinical Pathology and Medical Research, and Westmead Institute for Medical Research, The University of Sydney, Sydney, Australia; 18 Department of Neurology, University of Münster, Münster, Germany

**Keywords:** Myelin oligodendrocyte glycoprotein, Antibody, Epitope, antigen conformation, Optic neuritis, Multiple sclerosis, Diagnosis

## Abstract

Over recent years, human autoantibodies targeting myelin oligodendrocyte glycoprotein (MOG Ab) have been associated with monophasic and relapsing central nervous system demyelination involving the optic nerves, spinal cord, and brain. While the clinical relevance of MOG Ab detection is becoming increasingly clear as therapeutic and prognostic differences from multiple sclerosis are acknowledged, an in-depth characterization of human MOG Ab is required to answer key challenges in patient diagnosis, treatment, and prognosis. Herein, we investigated the epitope, binding sensitivity, and affinity of MOG Ab in a cohort of 139 and 148 MOG antibody-seropositive children and adults (*n* = 287 patients at baseline, 130 longitudinal samples, and 22 cerebrospinal fluid samples). MOG extracellular domain was also immobilized to determine the affinity of MOG Ab. MOG Ab response was of immunoglobulin G1 isotype, and was of peripheral rather than intrathecal origin. High affinity MOG Ab were detected in 15% paediatric and 18% adult sera. More than 75% of paediatric and adult MOG Ab targeted a dominant extracellular antigenic region around Proline42. MOG Ab titers fluctuated over the progression of disease, but affinity and reactivity to Proline42 remained stable. Adults with a relapsing course intrinsically presented with a reduced immunoreactivity to Proline42 and had a more diverse MOG Ab response, a feature that may be harnessed for predicting relapse. Higher titers of MOG Ab were observed in more severe phenotypes and during active disease, supporting the pathogenic role of MOG Ab. Loss of MOG Ab seropositivity was observed upon conformational changes to MOG, and this greatly impacted the sensitivity of the detection of relapsing disorders, largely considered as more severe. Careful consideration of the binding characteristics of autoantigens should be taken into account when detecting disease-relevant autoantibodies.

**Electronic supplementary material:**

The online version of this article (10.1186/s40478-019-0786-3) contains supplementary material, which is available to authorized users.

## Introduction

Myelin oligodendrocyte glycoprotein (MOG) is a myelin transmembrane protein exclusively expressed by oligodendrocytes within the central nervous system (CNS). The single extracellular MOG domain adopts a folded immunoglobulin (Ig) variable topology formed by two anti-parallel β-sheets surrounding a hydrophobic core [4, 7]. Localized on the outermost lamellae of CNS myelin, exposure to the extracellular space has placed MOG in the spotlight as a prime target of autoimmune demyelination [4, 17, 41]. Indeed, recent years have witnessed rapid expansion in the clinical spectrum of disorders associated with MOG antibodies (MOG Ab), and their detection is now implemented to diagnose patients with CNS demyelinating disorders phenotypically distinct from multiple sclerosis. The presence of MOG Ab has been most commonly associated with monophasic and recurrent episodes of optic neuritis (ON), acute disseminated encephalomyelitis (ADEM), and transverse myelitis (TM) in children, as well as unilateral ON, bilateral ON, and TM in adults [5, 6, 13, 23, 28, 35, 39, 42, 47, 75]. Although the clinical spectrum of MOG Ab has been well explored, an in-depth characterization of the human MOG Ab response is lacking and is required to broaden our biological understanding of MOG Ab-associated autoimmunity.

The introduction of the live cell-based assay, which retains MOG in its native form, has been seminal to establish the clinical relevance of MOG Ab. The progression to accurately detect disease-relevant MOG Ab has exposed technical caveats heavily rooted in the structural form and conformation of MOG. There is now a consensus that cell-based assays which retain MOG in its folded and inherent tertiary structure, rather than denatured or linearized, may enable accurate detection of human MOG Ab [74]. In parallel, numerous studies in animals have demonstrated that MOG Ab requires the recognition of a conformational epitope for antigen-Ab binding and to induce pathogenicity [3, 14, 58, 67, 68]. Investigations into the importance of antigen conformation on MOG Ab binding in large human cohorts has yet to be addressed and can provide valuable insights to improve MOG Ab detection for clinical diagnosis.

In addition to MOG conformation, epitope recognition and Ab binding affinity are of considerable importance. In children, the major conformational MOG Ab epitope includes Proline42 within the CC’-loop of the extracellular MOG domain [34], and a study of five adults has reported recognition of other extracellular epitopes, including Proline42 [57]. Recently, high affinity MOG Ab purified from two patients induced pathogenesis in two rat models [58]. Both high and low affinity autoantibodies have been shown to be pathogenic in rodent models of neuromyelitis optica (NMO) [31] and autoimmune hemolytic anaemia [16], respectively. However, antibody affinity remains to be defined in human MOG Ab-associated disorders, and larger MOG Ab epitope studies and their correlations with clinical phenotypes are warranted in both paediatric and adult demyelination.

Herein, we investigated the binding sensitivity, epitope reactivity, and affinity of human MOG Ab in a cohort of 287 paediatric and adult patients with MOG Ab-associated disorders. We defined the major MOG Ab response present in this large cohort and correlated antibody features with clinical phenotypes to improve diagnosis, relapse prediction, and ultimately patient treatment.

## Material and methods

### Study design

The objective of this study was to characterize the epitope, affinity, and sensitivity to conformational changes of human MOG Ab response in demyelination. Locally and internationally recruited patient sera were screened for antibodies targeting native MOG between 2011 and early 2018. We have identified 287 MOG Ab-positive (MOG Ab+) children (*n* = 139, < 18 years at disease onset) and adults (*n* = 148, > 18 years at disease onset). Among the patients for whom we had additional clinical information, disease duration (from onset to first serum tested/baseline sample) was a median 0 year, interquartile range (IQR) 0–0 years and mean 0.47 ± 1.24 years in children (*n* = 122), and median 0 year, IQR 0–0.1 years and mean 1.46 ± 3.5 years in adults (*n* = 108). Among these samples, 83% of paediatric and 69% of adult samples were collected at disease onset. One hundred thirty serial sera from 51 MOG Ab+ patients (19 children, 32 adults) and 22 CSF (10 children, 9 adults) were also collected. Clinical phenotypes were retrospectively collected between July 2018 and December 2018, and patients were reported as relapsing if relapses had occurred over the study time frame with a minimum disease duration of 6 months (Table 1). Experimental data in this study was obtained when MOG Ab+ patient sera were combined and retested as one cohort. A flow cytometry live cell-based assay (live flow assay) was used to detect presence of patient serum Ab against conformational native-MOG [1, 13, 49]. The epitope of MOG Ab was assessed using a P42S mutant, consisting of full-length human MOG with the proline at position 42 substituted for serine. Conformational changes in native-MOG were modelled using paraformaldehyde which is known to alter protein structure [37, 59], and binding to fixed MOG was assessed using flow fixed assay and a commercial fixed biochip assay, with samples blinded and independently performed by an accredited external pathology laboratory. Binding to the immobilized extracellular Ig-like domain of MOG (MOG^1–117^) enabled detection of high affinity Ab by ELISA. In all flow cytometry and ELISA experiments, samples were reported positive if they were above 99th percentile of the control range in at least two of three quality-controlled experiments.

**Table 1:** Clinical characteristics of native-MOG Ab+ demyelination patients

	Children			Adults			All patients		
	*N (% total)*	Female *F:M (ratio)*	Age *Median (IQR)* ^a^	*N (% total)*	Female *F:M (ratio)*	Age *Median (IQR)* ^a^	*N (% total)*	Female *F:M (ratio)*	Age *Median (IQR)* ^a^
All phenotypes (All)^b^	139 (All)	73:63 (1.2)	8 (5–12)	148 (All)	92:56 (1.6)	40 (30–54)	287 (All)	165:119 (1.4)	22 (8–43)
Monophasic course	79 (57)	39:40 (1)	7 (4–11)	57 (39)	37:20 (1.9)	43 (30–56)	136 (47)	76:60 (1.3)	14 (6–38)
Relapsing course	44 (32)	29:15 (1.9)	8 (6–13)	61 (41)	37:24 (1.5)	40 (33–51)	105 (37)	66:39 (1.7)	30 (10–44)
Unknown	16 (12)	5:8 (0.6)	9 (4–13)	30 (20)	18:12 (1.5)	39 (27–53)	46 (16)	23:20 (1.2)	25 (12–49)
ON	38 (27 of All)	26:12 (2.2)	10 (7–13)	91 (61 of All)	58:33 (1.8)	44 (33–55)	129 (45 of All)	84:45 (1.9)	34 (15–49)
Monophasic ON	22 (58)	14:8 (1.8)	9 (7–13)	43 (47)	29:14 (2.1)	46 (33–57)	65 (50)	43:22 (2.0)	32 (12–54)
Relapsing ON	16 (43)	12:4 (3.0)	11 (8–16)	48 (53)	29:19 (1.5)	40 (33–51)	64 (50)	41:23 (1.8)	34 (17–49)
BON	18 (47 of ON)	11:7 (1.6)	9 (6–14)	40 (44 of ON)	25:15 (1.7)	39 (33–55)	58 (45 of ON)	36:22 (1.6)	33 (13–49)
Monophasic BON	14 (78)	8:6 (1.3)	9 (5–13)	23 (58)	15:8 (1.9)	47 (31–57)	37 (64)	23:14 (1.6)	29 (10–49)
Relapsing BON	4 (22)	3:1 (3.0)	12 (6–17)	17 (43)	10:7 (1.4)	36 (33–51)	21(36)	13:8 (1.6)	34 (21–49)
UON	17 (45 of ON)	13:4 (3.3)	10 (9–13)	45 (49 of ON)	29:16 (1.8)	44 (32–54)	62 (48 of ON)	42:20 (2.1)	34 (17–50)
Monophasic UON	8 (47)	6:2 (3.0)	10 (8–13)	20 (44)	14:6 (2.3)	45 (32–59)	28 (45)	20:8 (2.5)	34 (14–54)
Relapsing UON	9 (53)	7:2 (3.5)	11 (8–15)	25 (56)	15:10 (1.5)	40 (32–53)	34 (55)	22:12 (1.8)	34 (17–50)
Relapsing ON mixed	3 (8 of ON)	2:1 (2.0)	10 (9–15)	6 (7 of ON)	4:2 (2.0)	49 (36–58)	9 (7 of ON)	6:3 (2.0)	39 (12–52)
ADEM	53 (38 of All)	5 (3–8)	5 (3–8)	1 (1 of All)	1:0 (−)	25 (−)	54 (19 of All)	28:26 (1.1)	5 (3–8)
Monophasic ADEM	42 (79)	20:22 (0.9)	4 (3–8)	–	–	–	42 (78)	20:22 (0.9)	4 (3–8)
Relapsing ADEM^c^	11 (21)	7:4 (1.8)	6 (5–8)	1 (100)	1:0 (−)	25 (−)	12 (22)	8:4 (2.0)	6 (5–9)
ON/TM	–	–	–	9 (6 of All)	5:4 (1.3)	54 (28–59)	9 (3 of All)	5:4 (1.3)	54 (28–59)
Monophasic ON/TM	–	–	–	3 (33)	2:1 (2.0)	54 (43–60)	3 (33)	2:1 (2.0)	54 (43–60)
Relapsing ON/TM	–	–	–	6 (67)	3:3 (1.0)	44 (25–58)	6 (67)	3:3 (1.0)	44 (25–58)
LETM	10 (7 of All)	3:7 (0.4)	11 (6–15)	8 (5 of All)	5:3 (1.7)	28 (24–33)	18 (6 of All)	8:10 (0.8)	18 (10–29)
Monophasic LETM	10 (100)	3:7 (0.4)	11 (6–15)	7 (88)	4:3 (1.3)	26 (23–31)	17 (94)	7:10 (0.9)	17 (9–26)
Relapsing LETM	–	–	–	1 (13)	1:0 (−)	34 (−)	1 (6)	1:0 (−)	34 (−)
Relapsing ADEM/ON	10 (7 of All)	4:5 (0.8)	6 (5–10)	–	–	–	10 (3 of All)	4:5 (0.8)	6 (5–10)
Monophasic CIS	1 (1 of All)	1:0 (−)	5 (−)	–	–	–	1 (1 of All)	1:0 (−)	5 (−)
Other^d^	5 (3 of All)	2:3 (0.7)	10 (6–15)	7 (5 of All)	3:4 (0.8)	32 (27–42)	12 (4 of All)	5:7 (0.6)	28 (15–40)
Phenotypes *n* < 3^e^	6 (4 of All)	5:2 (2.5)	8 (6–11)	9 (6 of All)	5:4 (1.3)	40 (33–44)	15 (5 of All)	10:6 (1.7)	25 (8–42)
Unknown Phenotype	16 (11 of All)	5:8 (0.6)	9 (4–13)	23 (16 of All)	15:8 (1.9)	43 (26–54)	39 (14 of All)	20:16 (1.3)	25 (12–49)

*ADEM* Acute disseminated encephalomyelitis, *BON* Bilateral optic neuritis, *CIS* Clinically isolated syndrome, *LETM* Longitudinally extensive transverse myelitis, *ON mixed* Combination of BON and UON, *ON/TM* Simultaneous ON and TM, *UON* Unilateral optic neuritis

^a^*IQR* Interquartile range

^b^Phenotype of baseline sample

^c^Relapsing ADEM is multiphasic ADEM according to [30]

^d^Other: uncommon and atypical MOG Ab-associated phenotypes, including monophasic CIS/LETM, seizures, cerebellar, brainstem, brainstem and headache and fatigue, acute psychosis, optic perineuritis

^e^Phenotypes with less than three patients; children, monophasic LETM/UON (1), monophasic short TM (1), relapsing BON/LETM (2), relapsing BON/LETM/ADEM (1), relapsing LETM/UON (1); adults, monophasic short TM (2), monophasic LETM/ADEM (1), monophasic LETM/brainstem (1), relapsing short TM (2), relapsing TM mixed (1), relapsing BON/LETM/ADEM (1), relapsing ON/brainstem/short TM (1)

### Patient serum and CSF samples

Patient clinical phenotypes were delineated based on discussion with their treating neurologists and are accurate characterizations of patients’ presentations (Table 1). Detailed clinical and radiological phenotyping was outside of the scope of this study, and we have not conferred neuromyelitis optica spectrum disorder (NMOSD) 2015 diagnosis on these patients [72]. MOG Ab-seropositive patients did not fulfil 2017 revised McDonald criteria [62]. Those classified as ADEM fulfilled Krupp et al. criteria [30]. Seropositivity of 23 children and 12 adults have been previously reported in [47]. Age-matched controls (24 children, 24 adults) were selected on the basis of clinical disease (general medical and non-inflammatory neurological disorders), and not on their negative serostatus. Previously archived MOG Ab seronegative (MOG Ab-) patients (24 children, 24 adults) were also tested. They included patients with monophasic and relapsing disorders not typically associated with MOG Ab, such as multiple sclerosis (MS, fulfilling 2017 revised McDonald criteria), and clinically isolated syndrome (CIS) other than optic neuritis (ON), and longitudinally extensive transverse myelitis (LETM) [23, 26, 49, 52]. All control sera and MOG Ab- sera remained below the positivity threshold, whilst MOG Ab+ patients laid consistently above threshold and had low intra-assay variability (Additional file 1: Table S1). MOG Ab titers were measured in 51 MOG Ab+ patients (19 children, 32 adults) from whom 130 serial samples were tested (54 paediatric samples, median follow-up 17.6 months, IQR 3.6–28.9; 76 adult samples, median follow-up 4.7 months, IQR 1.7–13.) (Additional file 1: Table S2). Intrathecal MOG Ab were detected in undiluted cerebrospinal fluid (CSF) as previously described [13]. We obtained 22 CSF from seropositive-MOG Ab+ patients (12 samples from 10 children, 10 samples from 9 adults). CSF controls (24 non-demyelination controls; 14 demyelination controls) were tested in parallel. Intrathecal production of MOG Ab was assessed by determining the MOG Ab index: MOG Ab index = $\frac{\mathrm{QMOG}\ \left(\mathrm{CSF}\ \mathrm{MOG}\ \mathrm{Ab}\ \Delta \mathrm{MFI}/\mathrm{Serum}\ \mathrm{MOG}\ \mathrm{Ab}\ \Delta \mathrm{MFI}\right)}{\mathrm{QIgG}\ \left(\mathrm{Total}\ \mathrm{CSF}\ \mathrm{IgG}/\mathrm{Total}\ \mathrm{serum}\ \mathrm{IgG}\right)}$. A MOG Ab index greater than four indicated intrathecal synthesis of as previously described [25, 50, 51]. Total IgG in patient CSF and serum was determined using a human IgG ELISA kit following manufacturer’s instructions (Immunology Consultants Laboratory, Inc.).

### Human MOG constructs

Serum IgG binding to full-length native human MOG α1 isoform (native-MOG), a native MOG mutant P42S (native-P42S), and an empty vector control (native-CTL) were assessed. Transduced stable human embryonic kidney (HEK293) cells lines were generated as previously described [13]. Site-directed PCR was used to mutate the Proline42 (P42) for serine of the extracellular monomer MOG domain (1–117aa) (native-MOG^1–117^), generating native-P42S^1–117^, which was used to confirm the secondary structure of native-P42S. Native-MOG^1–117^ and native-P42S^1–117^ was subcloned into the pESG-IBA144 Stargate Accepter vector (IBA Lifesciences). HEK293T cells were transfected with Lipofectamine 3000 (ThermoFisher Scientific), secreted monomers were then purified using HisTrap and StrepTrap HP columns (GE Healthcare Lifesciences) following manufacturer’s instructions. Following buffer exchange, protein yield was quantified with Pierce BCA protein assay kit (ThermoFisher Scientific). The secondary structure of native-MOG^1–117^ and native-P42S^1–117^ were determined using an Aviv 215S circular dichroism (CD) spectrometer (Aviv Biomedical Inc.). Far-UV circular dichroism (CD) spectra were measured over 200-260 nm with a cell path length of 0.1 cm at 0.26–0.43 mg/mL in PBS at 25 °C. The mean of four spectra were obtained and normalized to mean residue weight ellipticity units (θ_MRW_).

### Analysis of patient MOG Ab binding by flow cytometry

Flow cytometry cell-based assays were used to detect presence of patient serum Ab (1:50) against conformational native-MOG, native-P42S, and formaldehyde-fixed MOG (fixed-MOG). For the fixed flow assay, HEK293 cells were incubated with fresh 4% formaldehyde for 10 min at RT and washed twice prior to the addition of patient serum (1:50). After extensive washing of patient sera, live or fixed cells were incubated with AlexaFluor 647-conjugated anti-human IgG (H + L) (1:100, A21445) or anti-human IgM (1:100, A21249, ThermoFisher Scientific). Detection of native-MOG IgG1 Ab by live flow assay was similarly performed by which an unconjugated mouse anti-human IgG1 (1:100, MH1013, ThermoFisher Scientific) was added, then washed, and followed by AlexaFluor 647-conjugated anti-mouse IgG (H + L) (1:100, A31571, ThermoFisher Scientific).

Native-MOG and fixed-MOG Ab binding levels were obtained from the delta median fluorescence intensity (ΔMFI): ΔMFI = MOG MFI – CTL MFI. The positive threshold was set at three standard deviations (3SD) above the mean of age-matched controls. Samples were reported positive if they were above the threshold in at least two of three quality-controlled experiments. Patient MOG Ab binding was also assessed using fixed-MOG, P42S (fixed-P42S), and CTL (fixed-CTL) cell lines. The intra-assay variability was higher in the fixed flow assay than the live flow assay (Additional file 1: Table S1). P42S expression was controlled in each experiment using the chimeric 8-18C5 mAb (monoclonal anti-rat MOG with murine variable region and human IgG1 constant region, secondary antibody: AlexaFluor 647-conjugated anti-human IgG (H + L) at 1:100 (A21445 from ThermoFisher Scientific) as described for other mutant antigens [56]. Levels of MOG Ab binding to P42 (either native-P42 or fixed-P42) in patients were determined by the formula: P42 Ab = MOG ΔMFI - P42S ΔMFI. Control sera were similarly analysed and used to establish a control reference range by calculating the 3SD above and below the control P42 Ab mean. Patient MOG Ab were assigned as Proline42 binders (above mean + 3SD), other epitope (between mean-3SD and mean + 3SD), or Serine42 binders (below mean-3SD). Reported samples remained in the same category in at least two of three independent experiments. All flow cytometry data was acquired using the high-throughput system on the LSRII flow cytometer (BD Biosciences). Data was analysed using FlowJo v10 (TreeStar) software and Microsoft Excel. All imaging data was acquired on a TCS SP5 confocal microscope (Leica) with a 63X oil numerical aperture 1.4 objective lens (Westmead Imaging Facility). Data were analysed using ImageJ v1.46 software (NIH).

### Detection of patient MOG Ab by fixed biochip assay

Controls (10 children, 11 adults), native-MOG Ab- (12 children, 12 adults), and native-MOG Ab+ patients (65 children, 59 adults) were blinded and independently tested using the formaldehyde-fixed indirect immunofluorescence BioChip™ assay (fixed biochip assay) as per manufacturer’s instructions (Euroimmun). Similar numbers of low, mid, and high native-MOG Ab+ patients were randomly selected. The fixed biochip assay was independently performed by an accredited external pathology laboratory at NSW Health Pathology-ICPMR, which is the referral centre for neuronal antibody testing in NSW (average 8000 samples tested per year by Euroimmun assays). All samples were blinded and scored by two independent experienced investigators. Sera were reported negative or scored 1 to 4 if positive. Equivocal patients were re-blinded and independently retested. To avoid bias between assays, and in absence of diagnostic criteria for MOG Ab-associated disorders, sensitivity and specificity were determined using two groups of patients: monophasic and relapsing disorders with reported MOG Ab-association (such as ADEM, ON, BON, LETM) [23, 26, 46, 48, 49, 52, 70], and monophasic and relapsing disorders with not yet reported MOG Ab-association and disorders not associated with MOG Ab (MS, CIS other than ON, and general medical and non-inflammatory neurological disorders) [23, 26, 49, 52, 70].

### Detection of patient MOG Ab by ELISA

ELISAs were conducted to detect high affinity MOG Ab binding to the extracellular MOG domain. Purified native-MOG^1–117^ and native-P42S^1–117^ (10 μg/mL) was coated onto 96-well Nunc MaxiSorp™ plates (ThermoFisher Scientific) in coating buffer (0.1 M Na_2_CO_3_, 0.1 M NaHCO_3_, Milli-Q water, pH 9.6) overnight at 4 °C. Control wells were coated with bovine serum albumin (BSA) (10 μg/mL) to detect background sera binding. Wells were blocked (PBS, 1% BSA) for 3 h at RT, patient sera (1:50) was incubated for 2 h at RT, HRP-conjugated goat anti-human IgG (1:2000) (ThermoFisher Scientific) was added for 1 h at RT, followed by 3,3′,5,5′-tetramethylbenzidine (Sigma-Aldrich, USA) for 15 min at RT, and 1 M HCl was added to stop the reaction. Wells were washed thoroughly with PBS, 0.05% Tween20 following each incubation. Optical density (OD) values were obtained at 450 nm and were wavelength- and blank-corrected. MOG Ab binding to native-MOG^1–117^ was determined by the formula: native-MOG^1–117^ ΔOD = native-MOG^1–117^ OD – BSA OD. The positive threshold was set at 3SD above the control mean ∆OD . Native-MOG^1–117^ Ab titers were also independently assessed in 15 serum samples by ELISA, as described above.

### Statistical analysis

Graphs were generated using Prism v7.0a (GraphPad Software) and Adobe Illustrator CC 2015 (Adobe Systems). Statistics were only performed on phenotype groups with eight or more patients (Table 1). The Kruskal-Wallis test was used for continuous data comparisons and a chi-square (χ^2^) test for categorical comparisons. Correlation analyses and *R*^2^ values were generated using a linear regression model. Boxplots, bar graphs, age, and disease duration are expressed as median and IQR. One representative out of three independent flow cytometry and ELISA experiments is shown.

### Compliance with ethical standards

Human research ethics approvals (NEAF 12/SCHN/395) were granted by the individual ethics committees for the participating hospitals. Informed consent was obtained from patients or carers in the case of paediatric patients.

## Results

### Higher titers of MOG Ab are associated with bilateral optic neuritis

**Fig. 1:**
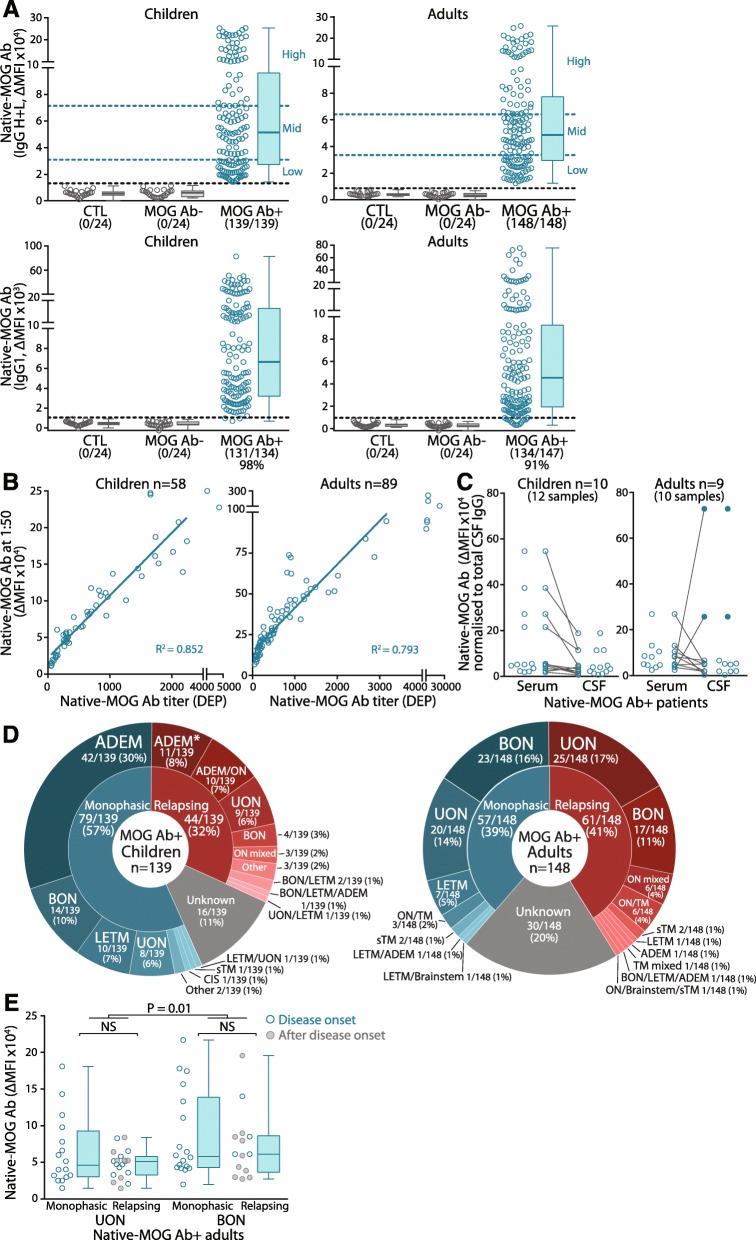
Human native-MOG Ab response in demyelinating disorders. **a** Paediatric (*n* = 139) and adult (*n* = 148) sera from patients with demyelinating disorders were positive for native MOG Ab by live flow assay (upper row). Most MOG Ab were of the IgG1 isotype (lower row). Based on level of native-MOG Ab (dotted blue line), patients had high (top 33% of all patients), mid (middle 33% of all patients), or low (lowest 33% of all patients) native-MOG Ab titers. All controls were negative (grey). Dotted-black line indicates positivity threshold (mean of control sera +3SD). Native-MOG Ab positivity is shown between brackets. **b** There was a strong positive correlation between the ΔMFI of serum diluted at 1:50 and MOG Ab titers represented by dilution end-point (DEP) in children (*n* = 58, *P* < 0.0001, *R*^2^ = 0.852) and adults (*n* = 89, *P* < 0.0001, *R*^2^ = 0.793). **c** CSF native-MOG Ab were detected in 10 seropositive children (12 samples) and 9 adults (10 samples). 10/12 paediatric and 8/10 adult CSF MOG Ab titers were lower than in matched serum (*P* = 0.111 and *P* = 0.429, respectively) when ΔMFI values were normalized. Two children had slightly elevated levels in CSF compared to serum, and two adults had significantly higher native-MOG Ab titers in CSF than serum (filled-blue). **d** Distribution of seropositive native-MOG Ab among paediatric and adult clinical phenotypes. Relapsing ADEM is multiphasic ADEM according to [30]. **e** Regardless of disease course, patients with BON had significantly higher titers of native-MOG Ab than UON patients (*P* = 0.01). BON patients also exhibited higher MOG Ab titers than UON patients when sera were collected at disease onset (monophasic and relapsing UON, *n* = 27; monophasic and relapsing BON *n* = 21) (*P* = 0.040). Ab- = antibody negative, Ab+ = antibody positive, ADEM = acute disseminated encephalomyelitis, relapsing ADEM* = multiphasic ADEM, BON = bilateral optic neuritis, CSF = cerebrospinal fluid, CTL = controls, DEP = dilution end-point, LETM = longitudinally-extensive transverse myelitis, MFI = median fluorescence intensity, sTM = short transverse myelitis, UON = unilateral optic neuritis

IgG antibodies targeting native MOG (native-MOG Ab) were detected in 139 children and 148 adult patients using a live flow assay (Fig. 1a) with low intra-assay variability (Additional file 1: Table S1). Native-MOG Ab-seropositive (native-MOG Ab+) patients exhibited a broad range of median fluorescence intensity (ΔMFI) values and were categorized into three levels of native-MOG Ab, with no significant differences between children and adults (Fig. 1a). Most native-MOG Ab (131/134 children, 98%; 134/147 adults, 91%) were of the IgG1 isotype (Fig. 1a), whereas only a minority was IgM and IgG double-positive (3/133 children, 2%; 10/148 adults, 7%) (Additional file 1: Figure S1A). There was no correlation between IgG and IgM values in all patients, suggesting no cross-reactivity (Additional file 1: Figure S1B). Sensitivity and specificity were high and similar between assays detecting total IgG and IgG1 (Table 2). ΔMFI values were strongly associated with native-MOG Ab titers in children and adults (Fig. 1b and Additional file 1: Figure S2). There was a high concordance of native-MOG Ab-positivity between matched serum and CSF samples, and native-MOG Ab were higher in serum than CSF after normalization to total IgG and serum dilution (8/10 children, 7/9 adults) (Fig. 1c). Four patients (2 children and 2 adults) had increased CSF titers compared to serum, however the two adults had intrathecal synthesis of MOG Ab as their MOG Ab index between intrathecal and extrathecal compartments exceeded four [25, 50] (Additional file 1: Table S3). Among native-MOG Ab+ children, ADEM was the most prevalent phenotype (38%, 53/139), and most exhibited a monophasic course (79%, 42/53) (Table 1, Fig. 1d), whereas in adults, optic neuritis (ON) was most common (61%, 91/148), and most presented with relapsing unilateral ON (UON) (27%, 25/91). Across all phenotypes, the disease course of children was predominantly monophasic (57%, 79/139), whereas a relapsing course was slightly more frequent in adults (41%, 61/148) (Table 1, Fig. 1d). Overall, ON was the major clinical phenotype across the cohort, and mainly occurred in isolation (45%, 129/287), but ON in combination with other demyelinating syndromes (including myelitis and ADEM) was also observed in a small group (9%, 26/287) (Table 1, Fig. 1d). Native-MOG Ab presented in patients between 1 to 80 years of age, predominantly between ages 3–7 in children, and 29–33 and 53–57 in adults (Table 1, Additional file 1: Figure S3A), with no correlation between native-MOG Ab titer and age. ON presentation in isolation were distributed across all ages, whereas ADEM and ADEM/ON was only observed in children, and ON/TM was only observed in adults (Table 1, Fig. 1d and Additional file 1: Figure S3B). Despite a slight female preponderance, native-MOG Ab titers did not differ across sex or paediatric phenotypes (Table 1). Among adults with ON, native-MOG Ab titers were higher in patients with bilateral ON (BON) compared to UON, independently of a monophasic or relapsing course (*P* = 0.01) (Fig. 1e), and despite disease duration, which was not statistically different between adults with UON (median 0 year, IQR 0–0.25 years; mean 1.32 ± 3.16 years, *n* = 35) and BON (median 0 year, IQR 0–0.88 years; mean 1.19 ± 2.95 years, *n* = 35) (*P* = 0.97). Furthermore, when patients with serum collected at disease onset were independently analyzed (monophasic and relapsing UON, *n* = 27; monophasic and relapsing BON *n* = 21), similar results were observed; BON patients exhibited higher MOG Ab titers than UON patients (Fig. 1e) (*P* = 0.040), suggesting that these results were not influenced by the timing of sample collection.

**Table 2:** Comparison of sensitivity and specificity of MOG Ab detection assays

	Total cohort		Limited cohort	
	Sensitivity % (CI)	Specificity % (CI)	Sensitivity % (CI)	Specificity % (CI)
Children	*N* = 171		*N* = 80	
Live flow assay IgG (H + L)^a^	89.7 (83–94)	97.1 (83.4–99.9)	87.9 (77–94.3)	100 (73.2–100)
Live flow assay (IgG1)^b^	89.8 (82.8–94.2)	94.3 (79.5–99)	88.3 (76.8–94.8)	100 (73.2–100)
Fixed flow assay^a^	50.7 (42.1–59.4)	100 (87.7–100)	43.9 (31.9–56.7)	100 (73.2–100)
Fixed biochip assay^a^	–	–	60.6 (47.8–72.2)	100 (73.2–100)
Adults	*N* = 172		*N* = 70	
Live flow assay IgG (H + L)^a^	93.9 (88–97.2)	97.5 (85.3–99.9)	92.3 (80.6–97.5)	100 (78.1–100)
Live flow assay (IgG1)^b^	87.7 (80.5–92.6)	97.5 (85.3–99.9)	81.5 (68.1–90.3)	95.5 (75.1–99.8)
Fixed flow assay^a^	52.3 (43.4–61)	100 (89.1–100)	50 (36–64)	100 (78.1–100)
Fixed biochip assay^a^	–	–	55.8 (41.4–69.3)	100 (56.1–100)

^a^Analysis included 134 children and 132 adults (total cohort), and 66 children and 52 adults (limited cohort) with reported MOG Ab-associated disorders, and 37 children and 40 adults (total cohort), and 14 children and 18 adults (limited cohort) with not yet reported MOG Ab-associated disorders and disorders not associated with MOG Ab

^b^Analysis included 127 children and 130 adults (total cohort), and 60 children and 54 adults (limited cohort) with reported MOG Ab-associated disorders, and 35 children and 40 adults (total cohort), and 14 children and 22 adults (limited cohort) with not yet reported MOG Ab-associated disorders and disorders not associated with MOG Ab. CI = 95% confidence interval. Groups are described in Materials and methods

### The most prevalent MOG Ab-associated phenotypes exhibit high sensitivity to conformational changes to MOG

**Fig. 2:**
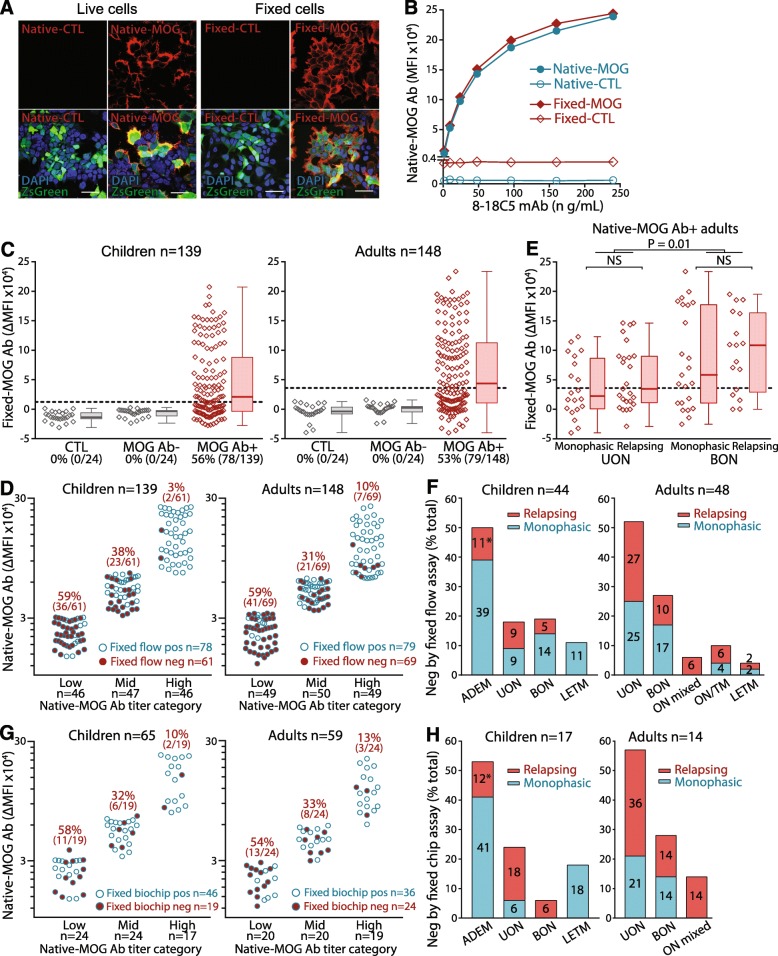
Human MOG Ab binding is influenced by conformational changes to native MOG. **a** and **b** High surface expression of native-MOG was comparable to formaldehyde-fixed MOG by immunocytochemistry (**a**, scale bar = 20 μm) and flow cytometry (**b**). **c** 78/139 (56%) native-MOG Ab+ children and 79/148 (53%) adult sera were positive for fixed-MOG Ab, whereas fixed-MOG Ab were not detected in all paediatric (*n* = 24) and adult (*n* = 24) control sera, and all native-MOG Ab- paediatric (*n* = 24) and adult (*n* = 24) sera (grey). Dotted line represents the positivity threshold (mean of controls +3SD). Fixed-MOG Ab positivity is shown between brackets. **d** In the fixed flow assay, 59% of children (36/61) and adults (41/69) who failed to bind fixed-MOG (filled-red) had low native-MOG Ab titers. A conformational insensitivity was observed in 10/46 children (22%) and 8/49 adults (16%) who could bind fixed-MOG despite low native-MOG Ab titers (empty-blue in low category). However, a sensitivity to conformation was seen in 25/61 (41%) children and 28/69 (41%) adults had mid to high native-MOG Ab titers. Number and percentage of patients negative in the fixed flow assay are shown in brackets. **e** Fixed-MOG Ab titers were higher in BON than UON patients (*P* = 0.01). **f** Adults seronegative by fixed flow assay predominantly presented with monophasic and relapsing UON (52%, 25/48), and BON (27%, 13/48), and 51% of non-binders had a relapsing course. Relapsing ADEM is multiphasic ADEM according to [30]. Unknown or other phenotypes (*n* = 16) and phenotypes with less than two patients are not represented (*n* = 5). **g** In the fixed biochip assay, reduced assay sensitivity was observed in 11/19 children (58%) and 13/24 adults (54%) who could not bind fixed-MOG in the fixed biochip assay (filled- red) and had low titers of native-MOG Ab. Conformational sensitivity was observed in 8/19 (42%) children and 11/24 adults (46%) who were negative by fixed biochip assay despite mid to high native-MOG Ab titers, and conformational insensitivity in 13/24 children (34%) and 7/20 (35%) adults positive in the fixed biochip assay but with low native-MOG Ab (empty-blue in low category). **h** Most adults seronegative by fixed biochip assay presented with monophasic and relapsing UON (57%), followed by BON (28%), and 64% had a relapsing course. Unknown phenotypes (*n* = 5) and phenotypes with less than two patients are not represented (*n* = 5). Relapsing ADEM is multiphasic ADEM according to [30]. θ_MRW_ = mean residue weight ellipticity, Ab = antibody, native-MOG^1–117^ = extracellular MOG, fixed-MOG = fixed MOG, fixed-CTL = fixed transduced control cells, MFI = median fluorescence intensity, UON = unilateral optic neuritis, BON = bilateral optic neuritis, ON mixed = combination of BON and UON, ON/TM = simultaneous ON and TM, LETM = longitudinally extensive transverse myelitis, relapsing ADEM* = multiphasic ADEM

Antigen conformation is known to influence Ab recognition, however, the effect of conformational changes to MOG on Ab binding remains elusive. Therefore, the sensitivity of human MOG Ab binding to fixed-MOG was assessed in formaldehyde-fixed flow cytometry and commercial biochip assays. Formaldehyde modifies proteins on various structural levels [37, 59], and with five primary amine groups within its extracellular domain, amine cross-linkage may not disrupt MOG secondary structure, but will change the native conformation of MOG. Expression of fixed-MOG was high and comparable to native-MOG (Fig. 2a and b), therefore fixed-MOG was available for binding by patient native-MOG Ab. Only 56% (78/139) of children and 53% (79/148) of adult native-MOG Ab+ sera were able to bind to fixed-MOG (Fig. 2c). The remaining native-MOG Ab+ patients (61/139 children, 44%; 69/148 adults, 47%), all control sera (48/48), and native-MOG Ab- sera (48/48) did not recognize fixed-MOG (Fig. 2c). 59% of patients who failed to recognize fixed-MOG had low native-MOG Ab titers (36/61 children; 41/69 adults) (Fig. 2d), whereas 41% of patients (25/61 children, 41%; 28/69 adults, 41%) did not recognize fixed-MOG despite having mid to high native-MOG Ab titers (Fig. 2d). However, 22% of children (10/46) and 16% of adults (8/49) bound fixed-MOG even with low native-MOG Ab titers (Fig. 2d). Native-MOG Ab titers correlated poorly with fixed-MOG Ab titers (Additional file 1: Figure S4A). These data suggest that failure of native-MOG Ab to bind fixed-MOG was influenced by two key features, which are not mutually exclusive: a low native-MOG Ab titer, and a MOG Ab response with sensitivity to native conformational MOG. Adults with monophasic and relapsing UON exhibited weaker binding to fixed-MOG than BON (*P* = 0.01, Fig. 2e) and were more likely to be seronegative in the fixed flow assay (*P* = 0.03, Fig. 2f) (Additional file 1: Table S4). Interestingly, adult females had weaker binding to fixed-MOG and were more likely to become seronegative as assessed by the fixed flow assay than adult males (*P* = 0.03).

We then examined whether this loss of binding to conformationally altered MOG was echoed in a formaldehyde-fixed biochip assay in a smaller cohort externally tested (65 children, 59 adults). Parallel to the fixed flow assay, a proportion of native-MOG Ab+ patients did not recognize fixed-MOG in the fixed biochip assay (29%, 19/65 children; 41%, 24/59 adults), and all controls (10 children, 11 adults) and all native-MOG Ab- patients (12 children, 12 adults) were negative (Additional file 1: Figure S4B). As in the fixed flow assay, binding to fixed-MOG was also dependent on native-MOG Ab category, as 58% of paediatric and 54% of adult patients who were negative had low native-MOG Ab titers (11/19 children; 13/24 adults) (Fig. 2g). Conformational sensitivity was observed in fixed-MOG Ab- patients with mid to high native-MOG Ab titers (42%, 8/19 children; 46%, 11/24 adults), whereas conformational insensitivity was observed in fixed-MOG Ab+ patients despite low native-MOG Ab titers (34%, 13/24 children; 35%, 7/20 adults) (Fig. 2g).

Between the fixed flow and biochip assays, 65% of children (30/46) and 71% of adults (25/35) were positive in both assays, with less overall seropositivity detected in the fixed flow assay. The score of fixed-MOG Ab, a qualitative measure of fluorescence intensity, in the fixed biochip assay correlated poorly to fixed-MOG Ab titers (Additional file 1: Figure S4C), implying a difference in the fixation effect on MOG between the fixed flow and biochip assays. Across the three assays, 30/66 (45%) children and 25/59 (42%) adults were positive. Overall, although the specificity of detection remained high between all assays, albeit slightly increased in the fixed flow and biochip assays, the sensitivity of the live flow assay was far greater than both fixed MOG assays (Table 2). Importantly, phenotypes which have recently been described as classical for MOG Ab-associated demyelination, such as children presenting with monophasic ADEM, UON, and BON, and adults presenting with relapsing UON and BON, were reported seronegative by fixed assays (Fig. 2f and h, Additional file 1: Table S4). A number of patients in whom detailed clinical information was available, and who tested seronegative by the fixed flow and biochip fixed assays, presented with typical MOG Ab-associated phenotypes and were therefore considered bona fide MOG Ab-seropositive patients by their clinicians rather than false positives (Additional file 1: Supplementary material 1). Furthermore, 19 children and 28 adults, in whom therapeutic data was available, were highly sensitive and responsive to immunotherapy, particularly steroids (Additional file 1: Table S4), once again highlighting that these patients behave in a manner considered typical for MOG Ab-associated demyelination.

### Reduced immunoreactivity to Proline42 is associated with relapsing optic neuritis

**Fig. 3:**
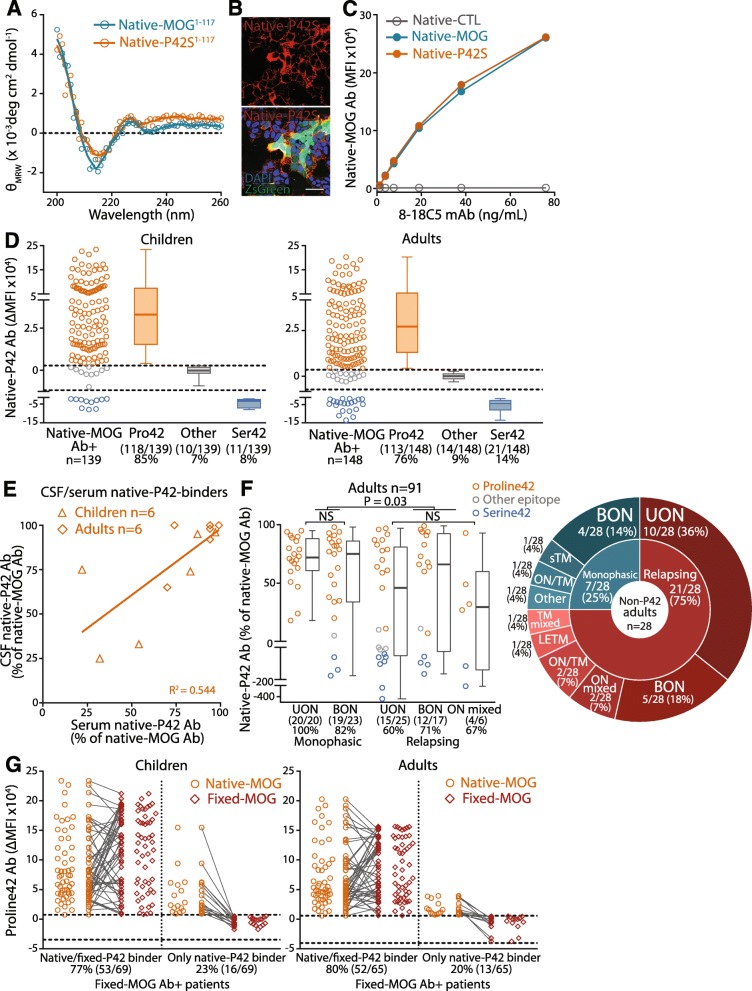
Proline42-binding MOG Ab are the dominant IgG in serum of children and adults with demyelinating disorders. **a** Circular dichroism spectra of extracellular MOG (native-MOG^1–117^) and extracellular P42S (native-P42S^1–117^) domains showed characteristic minima at 215 nm, indicating β-sheet folding in both antigens. **b** and **c** High surface expression of native-P42S was comparable to native-MOG by immunocytochemistry (**b**) and flow cytometry (**c**). Scale bar = 20 μm. **d** 118/139 children (85%) and 113/148 adults (76%) harbored native-P42 Ab (orange), whereas 11/139 children (8%) and 21/148 (14%) adults had native-S42 Ab (blue). 10/139 paediatric (7%) and 14/149 (9%) adult sera recognized an unknown epitope on native-MOG (grey). Dotted line represents the control reference range determined by age-matched controls (*n* = 24 children, *n* = 24 adults). Number and percentage of patients in each epitope category are shown. **e** There was a positive correlation between native-P42 Ab titers in CSF and matched-serum in six children (diamonds) and six adults (triangles) (*P* = 0.006, *R*^2^ = 0.545). **f** Most patients with monophasic UON and BON recognized native-P42 (100%, 20/20 and 83%, 19/23, respectively, orange), whereas relapsing ON patients had lower titers of native-P42 Ab and were more likely to recognize an epitope outside P42 (blue or grey, *P* = 0.03, left). Percentage and number of native-P42 binders in each phenotype group are shown. 75% of adult patients with an epitope outside P42 (non-P42, *n* = 28) presented with a relapsing course (right). **g** Among patients with native-P42 and fixed-MOG Ab, most paediatric and adult sera recognized the P42 epitope in native-P42 and fixed-P42 conditions (77%, 53/69 children; 80%, 52/65 adults), whilst a group lost their binding to the P42 epitope when MOG was fixed (23%, 16/69 children; 20%, 13/65 adults). θ_MRW_ = mean residue weight ellipticity, Ab = antibody, BON = bilateral optic neuritis, CSF = cerebrospinal fluid, MFI = median fluorescence intensity, native-MOG^1–117^ = extracellular MOG, native-MOG = native human MOG, native-CTL = native transduced control cell, native-P42 Ab = native Proline42 binding MOG Ab, ON mixed = combination of BON and UON, S42 = Serine42, UON = unilateral optic neuritis

To determine whether Proline42 was included in the major antigenic region as reported in children [34], MOG Ab binding was compared between P42S MOG (native-P42S) and native-MOG in our large paediatric and adult cohort. The secondary protein structure of native extracellular P42S MOG (native-P42S^1–117^) was not altered by the point mutation, with the circular dichroism (CD) spectrum characteristic of β-sheet folding and similar to extracellular MOG (native-MOG^1–117^) (Fig. 3a). Furthermore, surface expression of native-P42S was high and comparable to native-MOG (Figs. 2e and 3b and c), which enabled reliable assessment of binding between native-P42S and native-MOG. Proline42 within the extracellular CC’ loop of native-MOG was crucial to the binding of most human native-MOG Ab+ patients: 85% of children (118/139) and 76% of adults (113/148) (Fig. 3d). The epitope was not identified in a small group who bound similarly to native-P42S and native-MOG (7%, 10/139 children; 9%, 14/148 adults), and a minor group presented with strong immunoreactivity to native-P42S, and therefore bound Serine42 (8%, 11/139 children; 14%, 21/149 adults) (Fig. 3d). Proline42 was an immunodominant epitope, with more than 75% of native-MOG Ab targeting P42 (Additional file 1: Figure S5A). Native-P42 Ab titers and percentage of total MOG Ab response between paediatric and adult sera were similar (Fig. 3d and Additional file 1: Figure S5A). 67% (8/12) of paediatric and 70% (7/10) of adult CSF MOG Ab recognized native-P42. When matched CSF and serum samples were compared, 75% (6/8) of children and 86% (6/7) of adult CSF P42-binders also had serum responses toward Proline42, and the percentages of native-P42 Ab in serum and CSF were correlated (Fig. 3e), suggesting no major change in epitope across intrathecal and peripheral native-MOG Ab responses.

Across all adult patients, reactivity to native-P42 was significantly lower in relapsing phenotypes than monophasic syndromes (*P* = 0.015). Compared to adults with monophasic ON (UON and BON), relapsing ON patients had lower immunoreactivity to native-P42S (*P* = 0.014, Additional file 1: Figure S5B), and a lower percentage of native-P42 Abs (*P* = 0.038, Fig. 3f) with more patients recognizing other epitopes or Serine42, regardless of lesion localization, i.e. bilateral or unilateral. These results show that 34% of relapsing adults (35% of relapsing ON including ON mixed) intrinsically present with a more diverse MOG Ab response characterized by binding of additional epitopes not affected by the P42S mutation (Fig. 3f). Overall, 75% of adult patients with an epitope outside Proline42 presented with a relapsing course (Fig. 3f). No statistical differences were found across paediatric phenotypes.

To assess whether conformational change prevented binding of native-MOG Ab to its epitope, we compared patient recognition of Proline42 between native-P42 and fixed-P42. Of those who bound fixed-MOG, 69/78 children (88%) and 65/79 adults (82%) recognized native-P42. Within these P42-binders, most patients recognized the same Proline42 epitope across the two assays (77%, 53/69 children; 80%, 52/65 adults) (Fig. 3g). However, 16/69 children (23%) and 13/65 adults (20%) who bound native-P42 lost their recognition of Proline42 on fixed-MOG, suggesting that the conformational change abrogated binding to their natively-presented Proline42 epitope (Fig. 3g).

### MOG Ab titers fluctuate over time and Proline42 binders have stable immunoreactivity in serum

**Fig. 4:**
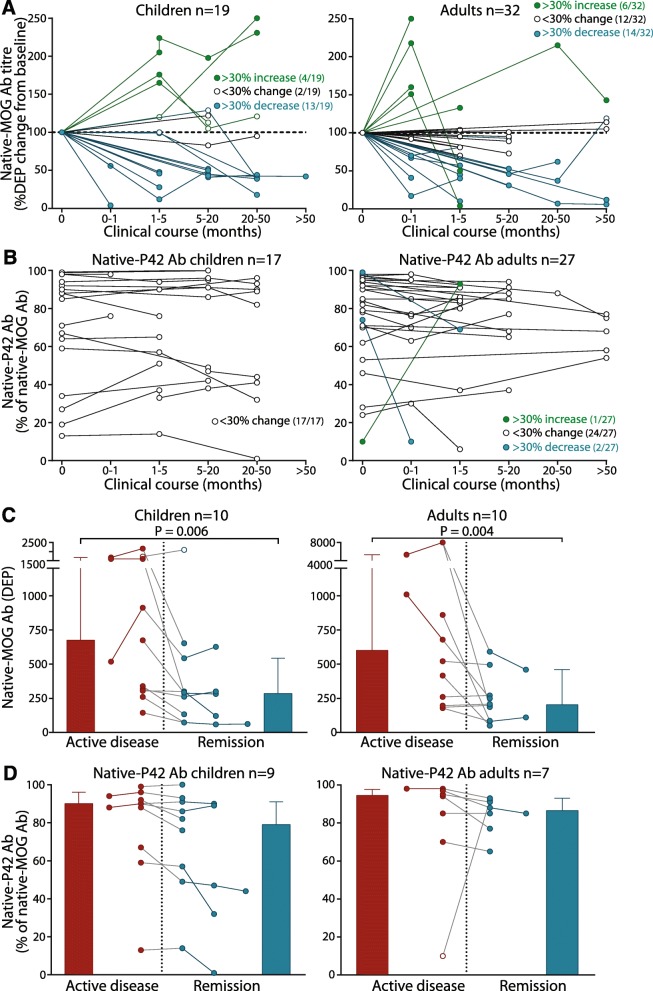
The titer, but not epitope, of human MOG Ab changes over time. **a** Compared to the sample at baseline, MOG Ab titers, represented by DEP, decreased by more than 30% (blue-filled) in 13/19 paediatric (68%) and 14/32 adult sera (44%), and increased by more than 30% (green-filled) in 4/19 children (21%) and 6/32 adults (19%) for up to 4.7 years (median 17.6 months, IQR 3.6–28.9) and 9.1 years (median 4.1 months, IQR 1.7–13.9), respectively. Dotted lines represent native-MOG Ab titer (100%) at the baseline sample of each patient. **b** Native-MOG Ab immunoreactivity toward P42 did not change regardless of initial native-P42 Ab titers in all 17 paediatric and 24/27 adult (89%) native-P42 Ab-seropositive patients for up to 4.7 and 9.1 years, respectively. Two adults with high titers of native-P42 Ab decreased (blue), and one adult developed high immunoreactivity to native-P42 (green). **c** and **d** Sera collected during active disease (*n* = 13 paediatric and *n* = 12 adult samples, red) and disease remission (*n* = 15 paediatric and *n* = 12 adult samples, blue) were available in 10 children and 10 adults. **c** Native-MOG Ab titers were higher during acute samples than remission samples (*P* = 0.006, children; *P* = 0.004, adults). **d** Among 9 children and 7 adults recognizing native-P42, 8/9 children and 6/7 adults had slightly weaker immunoreactivity to native-P42 during remission (children, *P* = 0.317; adults, *P* = 0.226). Ab = antibody, DEP = dilution end-point, native-MOG = native human MOG, native-P42 Ab = native Proline42 binding MOG Ab

Longitudinal analysis of native-MOG Ab titers was examined in 130 native-MOG Ab+ samples from 51 patients (Additional file 1: Table S2). The majority of patients longitudinally tested had a relapsing course (84%, 16/19 children; 69%, 22/32 adults). Most children presented with monophasic and relapsing ADEM (5/19 in isolation, 5/19 in combination with other syndromes) and ON (3/19 BON, 3/19 UON, 3/19 ON/LETM), whereas most adults presented with ON (13/32 BON, 9/32 ON) (Additional file 1: Table S2). From baseline, represented by the first collected sample, most patients (89%, 17/19 children; 63%, 20/32 adults) had fluctuating native-MOG Ab titers over time, spanning up to 4.7 years in children (median disease duration 17.6 months (interquartile range (IQR) 3.6–28.9) and 9.1 years in adults (median disease duration 4.1 months (IQR 1.7–13.9) (Fig. 4a). The majority of patients, 13/19 children and 14/32 adults, had at least 30% decrease in native-MOG Ab titer from baseline (Fig. 4a), whereas a smaller proportion of 4/19 children and 6/32 adults had a greater than 30% increase of MOG Ab titers. 2/19 children and 12/32 adults showed stable MOG Ab titers over time (Fig. 4a). In this cohort, patient phenotype did not predict an increase or decrease in MOG Ab titer. Persistent levels of native-MOG Ab, reported when patients maintained MOG Ab-seropositivity for at least 3 months, was observed in 15/19 children (79%) and 20/32 adults (63%), with the majority exhibiting a relapsing phenotype (80%, 12/15 children; 70%, 14/20 adults) (Additional file 1: Table S2). Most longitudinal patients harbored native-P42 Ab at baseline (89%, 17/19 children; 84%, 27/32 adults). In all 17 children and most adults (89%, 24/27), the percentage of native-P42 Ab among total native-MOG Ab remained stable across their disease course regardless of their initial native-P42 Ab percentage (Fig. 4b). Longitudinal stability was also observed when native-P42 Ab titer fluctuations paralleled native-MOG Ab titer changes over time (Additional file 1: Figure S5C and D). Two adults had reduced percentage of native-P42 Ab whereas another adult had an increase in percentage of native-P42 Ab (Fig. 4b and Additional file 1: Figure S5E). However, their native-MOG Ab titers fluctuated significantly resulting in the observed large native-P42 Ab percentage change (Additional file 1: Figure S5F).

Native-MOG Ab titers were higher during active disease and decreased during disease remission in all 10 children and 10 adults (children, *P* = 0.006; adults *P* = 0.004) (Fig. 4c). Interestingly, although there was stable native-P42 Ab across disease course, the percentage of native-P42 Ab was slightly higher during active disease than at remission in 9/9 children and 6/7 adults (*P* > 0.05) (Fig. 4d). One adult, who harbored a low percentage of native-P42 Ab during active disease, had a significant increase of native-P42 Ab during remission (Fig. 4d), however this patient had a 10-fold decrease of native-MOG Ab titres from active disease to remission (Fig. 4c and Additional file 1: Figure S4, C and D, Patient A).

### MOG Ab with high immunoreactivity to the immobilized extracellular MOG Ig-like domain exists in a small population of patients

**Fig. 5:**
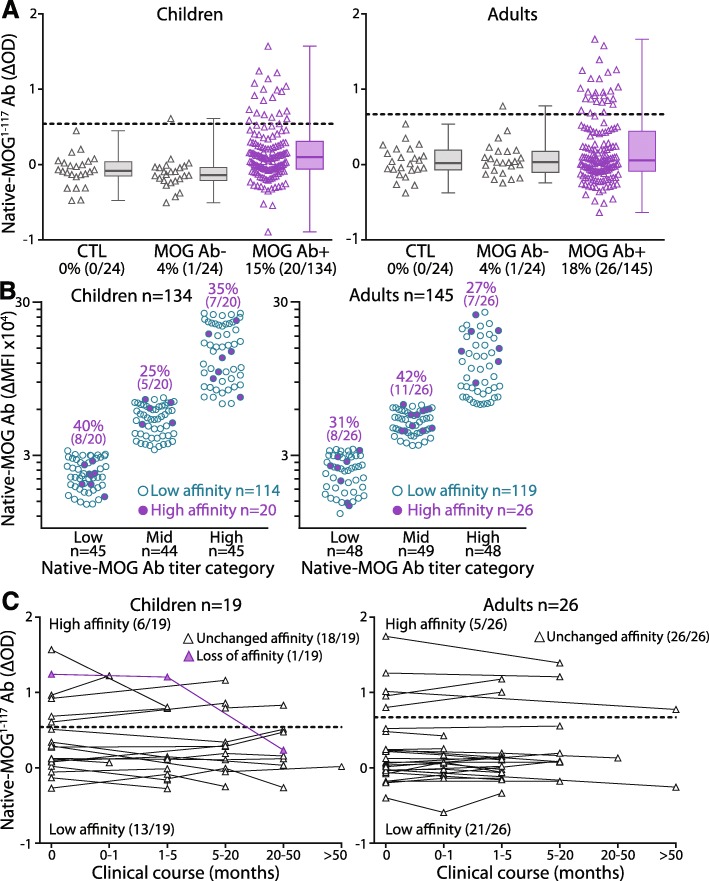
A small population of children and adults harbor high affinity MOG Ab. **a** 20/134 children (15%) and 26/145 adult sera (18%) bound to native-MOG^1–117^ and had high affinity MOG Ab, whereas no controls (grey), and one native-MOG Ab- child and one adult were positive for native-MOG^1–117^ Ab (grey). Dotted line represents the positivity threshold and native-MOG^1–117^ Ab positivity is shown between brackets. **b** Presence of high affinity native-MOG^1–117^ Ab (purple) were not associated with native-MOG Ab titers, with similar detection of high affinity MOG Ab (purple) in children and adults across low, mid, and high native-MOG Ab titers. **c** High affinity MOG Ab was sustained for up to 1.6 years in 5/6 children (median disease duration 3.4 months, IQR 1.6–5.8) and 4.9 years in all five adults (median disease duration 5.1 months, IQR 4.2–13.7), whereas high affinity MOG Ab disappeared in one child at 20.8 months. Ab- = antibody negative, Ab+ = antibody positive, CTL = controls, native-MOG^1–117^ Ab = high affinity MOG Ab binding to extracellular MOG, native-MOG = native human MOG, OD = optical density

Overexpression of antigens in the live flow assay permits high antigen density and oligomerization of cell surface MOG that enables detection of high and low affinity antibodies [33, 58, 69]. A recent study detected high affinity MOG Ab using an immobilized folded extracellular MOG domain [58]. To a similar extent, we immobilized the extracellular domain of MOG spanning amino acids 1–117 (native-MOG^1–117^) to detect high affinity MOG Ab by ELISA. 15% of children (20/134) and 18% of adults (26/145) bound to native-MOG^1–117^, and therefore harbored high affinity MOG Ab (Fig. 5a). No controls were above the positive threshold, however, among the native-MOG Ab- patients, one adult and one child exhibited low levels of native-MOG^1–117^ Ab. The Δ optical density (OD) values were positively correlated with native-MOG^1–117^ Ab titer (Additional file 1: Figure S6A), suggesting a higher ΔOD value denoted a higher Ab concentration. The presence of high affinity MOG Ab was observed among a range of native-MOG Ab titers (Fig. 5b). Most native-MOG^1–117^ Ab recognized native-P42^1–117^ (55%, 11/20 children; 77%, 20/26 adults) and most of these patients (55%, 6/11 children; 70%, 14/20 adults) similarly harbored native-P42 Ab (Additional file 1: Figure S6B). There was no correlation between native-MOG^1–117^ and native-MOG Ab titers (Additional file 1: Figure S6C), suggesting these patients have a combination of high and low affinity MOG Ab. Native-MOG^1–117^ and fixed-MOG Ab titers were compared to determine whether high affinity MOG Ab allowed binding to fixed-MOG, and no correlation was observed (Additional file 1: Figure S6C), suggesting high binding in the live and fixed flow assays was not dependent on Ab affinity.

Presence of high affinity MOG Ab was sustained for up to 1.6 years in 5/6 children (median 3.4 months, IQR 1.6–5.80) and up to 4.9 years in all adults (5/5, median 5.1 months, IQR 4.2–13.7) (Fig. 5c). High affinity MOG Ab disappeared in one child at 20.8 months (Fig. 5c) which also coincided with an 80% decrease in native-MOG Ab. High affinity MOG Ab did not appear throughout clinical course in any patients with low affinity Ab at baseline, up to 4.7 years in children and 9.1 years in adults. In line with native-MOG Ab responses, all patients who recognized native-P42^1–117^ maintained the same epitope recognition throughout their disease course (4 children, 4 adults) (Additional file 1: Figure S6D). Children with high affinity MOG Ab predominantly presented with ADEM (55%, 7/20 monophasic and relapsing ADEM, and 4/20 ADEM/ON), whereas adults with high affinity MOG Ab mainly had BON (30%, 8/26, monophasic and relapsing) followed by UON (24%, 6/26, monophasic and relapsing, Additional file 1: Figure S6E). Monophasic patients were more likely to harbor low affinity MOG Ab than relapsing patients (*P* = 0.088).

### Most human MOG Ab responses recognize Proline42 with low affinity and monophasic patients are more insensitive to conformational change

**Fig. 6:**
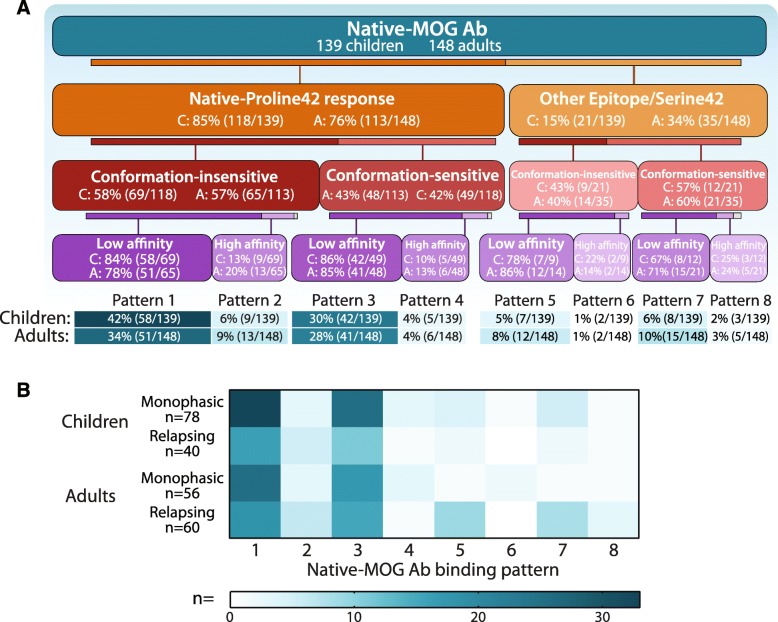
Major MOG Ab binding Patterns in children and adults with demyelinating disorders. **a** Native-MOG Ab binding was divided into eight patterns characterized by Proline42 epitope (orange), conformational sensitivity (red), and affinity (purple). MOG Ab responses from paediatric (42%) and adult (34%) sera targeted P42 and included conformation insensitive low affinity Ab (Pattern 1), followed by 30% of children and 28% of adults with similar low affinity response against Proline42, but were conformation-sensitive (Pattern 3). Responses targeting an epitope outside of Proline42 (light-orange) were of low affinity but were more sensitive to conformational changes to MOG (lighter red). Color intensity decreases with lower frequency of patients in each category (5 children and 3 adults not tested for affinity). **b** Association between clinical course and MOG Ab binding Patterns. Adults with a monophasic disease course were likely to present Pattern 1 (*P* = 0.028). Patterns 5 and 7 were more likely to include adults with a relapsing course (*P* = 0.035). Scale bar denotes frequency of patients in each binding Pattern. Affinity was not assessed in 2 monophasic (1 child, 1 adult), 5 relapsing patients (4 children, 1 adult), and one adult with unknown phenotype. Ab = antibody

Eight MOG Ab binding patterns were identified based on their epitope, and binding to fixed-MOG and native-MOG^1–117^ (Fig. 6). Overall, the human native-MOG Ab response recognized an immunodominant epitope at Proline42, and comprised of conformation-insensitive low affinity Ab, more so in children than in adults (Pattern 1; 42%, 58/139 children; 34%, 51/148 adults) (Fig. 6a). The second-most prevalent MOG Ab response was conformation-sensitive and similarly targeted Proline42 with low affinity (Pattern 3; 30%, 42/139 children; 28%, 41/148 adults). Interestingly, when the response targeted an epitope outside Proline42, although the response was predominantly of low affinity, a sensitivity to conformation was more prevalent (Pattern 7 and 8 combined; 11/139 children, 8%; 20/148 adults, 13%) (Fig. 6a). When clinical phenotypes were analyzed, adults with a monophasic disease course were more likely to have MOG Ab binding Pattern 1, with recognition of Proline42, the ability to bind fixed-MOG, and failure to bind to native-MOG^1–117^ (*P* = 0.028) (Fig. 6b), whereas Patterns 5 and 7, characterized by low affinity Ab and binding to an epitope outside Proline42, were more likely to include adults with a relapsing course, irrespective of conformational sensitivity (*P* = 0.035) (Fig. 6b). There was no segregation of MOG Ab binding patterns between paediatric phenotypes.

## Discussion

The current study provides an in-depth characterization of the human MOG Ab response in a large cohort of paediatric and adult demyelinating disorders. The typical human MOG Ab is of low affinity and targets an extracellular epitope at Proline42. Ab binding requires native MOG conformation for many patients. High titers of MOG Ab are associated with more severe phenotypes of adult ON as defined by bilaterality of symptoms, and MOG Ab titers fluctuate over the progression of disease, with higher levels during active disease. The human MOG Ab response is highly confined to Proline42 with stable immunoreactivity over time and across peripheral and intrathecal compartments. Relapsing disorders present with a more diverse Ab repertoire, a feature that could be harnessed for patient management. MOG Ab are highly sensitive to conformational changes of MOG, which affect the detection of a substantial number of relapsing phenotypes, largely considered as more severe.

MOG Ab-associated disorders have a slight female predominance and appear between 1 and 80 years of age with seropositivity rates highest among children and young- and middle-aged adults. The clinical distribution in our cohort was similar to previous reports [6, 10, 19, 24–26, 47] with predominant presentation of ADEM among children, and ON across all ages. Consistent with previous studies [20, 26], most children presented with a monophasic disease course, whereas most adult patients exhibited a relapsing course. We found monophasic and relapsing ADEM and ADEM/ON to be more common in children [73], whereas monophasic and relapsing ON and ON/TM were dominant in adults. Clinical ADEM was rarely observed in adults. These differences in disease course and phenotype suggest a dichotomy in paediatric and adult MOG Ab-associated disorders, although we did not observe any significant difference in MOG Ab characteristics between these two groups. A recent study observed higher MOG Ab titers associated with increased visual and motor disability but did not correlate this data to a specific clinical phenotype [9]. By stratifying unilateral and bilateral ON patients, we showed that patients with the more severe phenotype of bilateral ON had higher MOG Ab titers and a greater binding to conformationally altered MOG, regardless of monophasic or relapsing disease course and disease duration. An association between high titers and ADEM in children [20, 23, 42] was not observed, possibly due to the preponderance of ON patients with high titers among our paediatric cohort. Furthermore, we showed elevated MOG Ab titers during active disease compared to disease remission, as previously reported [8, 20, 25]. Observations of high titers in more severe phenotypes and active disease directly support the pathogenic potential of MOG Ab in human demyelination.

Failure of the autoantibody to bind to its fixed antigen was influenced by lower native-MOG concentration and by a high sensitivity to conformational change. MOG Ab positivity from the fixed assays had high specificity, with no false-positivity, unlike a recent report in a larger control cohort [70]. However, the fixed flow and commercial assays had lower detection sensitivity and higher intra-assay variability compared to the live flow assay, with loss of seropositivity observed among monophasic but more so in relapsing patients. Furthermore, the patients undetected by fixed assays, presented clinical and radiological features typical of previously reported MOG Ab-associated disorders [5, 13, 20, 26, 27, 43, 47, 53], with many responding well to immunotherapy and relapsing upon steroid cessation. We therefore conclude that these patients were not false-positive patients in the live flow assay but were highly typical for MOG Ab-associated disorders. Formaldehyde has been known to alter protein structure and affect antibody recognition [37, 59, 61], but this caveat remains frequently overlooked in the context of the commercialisation of autoantibody detection, mostly due to pragmatic considerations including ease of performance. The crux in advancing our understanding of this disease entity relies entirely on accurate detection of seropositive MOG Ab patients. Given its clinical utility and the apparent higher incidence and prevalence of MOG Ab-associated demyelination compared to aquaporin-4 (AQP4) Ab-associated neuromyelitis optica spectrum disorders (NMOSD), requests for MOG Ab screening has dramatically risen. As the need for clinical distinction from other demyelinating disorders such as multiple sclerosis or NMOSD is essential due to therapeutic and prognostic implications [11, 45], the failure to detect these patients accurately is of significant concern.

Our data raises the question as to why human MOG Ab binding is so sensitive to antigen conformation. As the MOG extracellular domain contains five lysine residues, which are major reactive sites of cross-linking modifications [61, 63, 66], formaldehyde fixation is likely to distort individual β-strands to disrupt the antigen-antibody interaction of MOG Ab. In principle, fixation rigidifies protein structure, which will restrict protein flexibility to form appropriate epitope contact residues and also limit antigen reconfiguration which occurs to strengthen antibody-antigen contact [76]. Therefore, as the human MOG Ab response is highly dependent on an immunodominant region at Proline42, conformational changes to MOG will significantly affect MOG Ab binding. The Ab affinity, concentration, or recognized epitope did not define whether a patient was insensitive or sensitive to conformational changes to MOG. Upon formaldehyde fixation, the co-occurrence of buried epitope recognition sites, and exposure of natively-hidden or intracellular neoepitopes due to permeabilization, may play a role. Although the extent of antigen masking by fixation varies between proteins [54, 63], the caveats of formaldehyde fixation should still be carefully addressed in the context of other human autoantibodies in future diagnostic and functional assays. It is recommended that detection methods retain live and conformationally-correct MOG to maximise assay sensitivity and to ensure accurate detection of MOG Ab. Whether these conformation-insensitive MOG Abs have greater pathogenic potential than those sensitive to structural changes could be addressed in future.

Most children in our cohort recognized a conformational epitope at Proline42 within the extracellular CC’ loop, concurrent with a previous report [34]. In rodents, Proline42 is replaced by Serine42. A study on five MOG Ab seropositive adult patients with multiple sclerosis demonstrated the recognition of varying epitopes between all patients [57]. In this study, which is the largest to date, the majority of adult MOG Ab exhibited an immunoreactivity to Proline42, similar to children. Previous studies have observed that 49–60% of human MOG Ab sera were reactive to rodent brain tissue using immunohistochemistry, however, these studies demonstrated a broad reactivity against rodent brain antigens, and not specifically to rodent MOG [40, 55]. Our data in a mutant-expressing cell-based assay show a small percentage were able to bind P42S in which human Proline42 was replaced by rodent Serine42. Mayer et al. (2013) reported limited paediatric MOG Ab reactivity to mouse MOG-expressing cells which is concordant with our findings, as antibodies targeting Proline42 epitope represented a major antibody species among the total human MOG Ab response. Relapsing adults with ON were less reactive to Proline42 and more likely to recognise other epitopes than monophasic patients, suggesting a more diverse repertoire of the MOG Ab response. Instead, adults with relapsing ON were highly reactive to the generated rodent neo-epitope, Serine42, in the P42S mutant MOG. As relapses have been associated with increased disability over time [18, 47], early biomarkers of relapses are clinically useful and could alleviate the key challenge of predicting disease severity at onset. Among patients with an epitope outside Proline42, 75% had a relapsing course. Therefore, as P42S immunoreactivity remains stable over time, high binding to the P42S MOG mutant could be utilised to predict a relapsing course in adults at any stage of their disease. Interestingly, at an individual level, the proportion of Proline42 MOG Ab were slightly elevated during active disease compared to remission, which suggest Proline42 MOG Ab may contribute to disease pathology. Indeed, assessment of MOG Ab pathogenicity in rodent models will be important to understand disease pathogenesis. EAE, the classical rodent model for multiple sclerosis, could better reflect the neuropathology of human MOG Ab-associated disorders [32], however consideration of the major human epitope of MOG Ab should be prioritised in future in vivo studies.

The concept of epitope spreading has been reported in multiple sclerosis [44, 65], AQP4 Ab in NMO [64], but not in MuSK Ab-associated myasthenia gravis [21], nor in 11 paediatric MOG Ab responses [34]. At a cohort level, the proportion of Proline42 MOG Ab did not differ between adults and children. Furthermore, despite fluctuation of MOG Ab titers over time and baseline Proline42 Ab titers, the proportion of response toward Proline42 remained unchanged, even after 9 years, strengthening the assertion of stable epitope immunoreactivity of MOG Ab across child- and adult-hood. Additionally, the antibody specificity did not vary between peripheral blood and CSF which could be due to antibody diffusion after blood brain barrier impairment [38], which is also supported by the lack of an intrathecal synthesis of MOG Ab in our patients. Although the current study is limited to one epitope, Proline42 reactivity involved a large proportion of the MOG Ab response and remained unchanged, indicating little evidence of intramolecular spreading throughout a patient’s disease course, which is promising for epitope directed therapy.

The use of ELISAs to diagnose MOG Ab seropositive patients has been extensively discussed [15, 52, 71]. Although our results suggest that ELISAs are not useful to diagnose MOG Ab seropositivity compared to a live cell-based assay, however, once an intact extracellular MOG domain is utilized, quantification of antibody binding by ELISA remains appropriate, for example, to identify MOG Ab that are highly reactive to MOG. Likewise, in myasthenia gravis, ELISAs have been shown to detect high affinity Ab to the acetylcholine receptor [33, 76]. Indeed, conformational MOG epitopes remain available for binding in solid-phase assays such as ELISAs [36], and a recent study reported ELISA-positive patient MOG Ab targeting the extracellular MOG domain and binding to the same extent to the high affinity monoclonal 818C5 Ab [58]. We observed intact β-sheets of the MOG extracellular domain and detected presence of high affinity MOG Ab in a small population of MOG Ab-seropositive patients, a low incidence parallel to Spadaro et al. (2018). Furthermore, presence of high affinity MOG Ab did not determine whether a patient could bind fixed MOG or their MOG Ab titer, suggesting these antibodies comprise a small proportion of the total MOG Ab response. Although, both high [16] and low [33] affinity autoantibodies have been shown to induce pathogenicity, interestingly, in the case of MOG Ab, only high affinity human Ab, purified with a construct similar to ours, have been pathogenic in animal models so far [58]. Although we used the immunoreactivity to the immobilized MOG extracellular Ig-like domain to determine Ab affinity, direct evidence of affinity in human serum cannot be assessed due to unknown titers of peripheral MOG-specific antibody and probable polyclonality. As individual effects of high and low affinity antibodies cannot be distinguished in polyclonal serum, studies using patient-derived recombinant monoclonal MOG Ab are necessary to discriminate the pathogenic potential of high and low affinity MOG Ab. A relatively small percentage of patients had high affinity MOG Ab that persisted over time, even after 1.6 and 4.9 years in children and adults, respectively. Two patients with high affinity serum MOG Ab presented with intrathecal MOG Ab. These Ab may originate from peripheral antibody-secreting cells after post-germinal centre affinity maturation which then transit into the CNS as seen in AQP4 Ab-associated NMOSD and MuSK Ab-associated myasthenia gravis [2, 29, 60]. On the other hand, lower affinity MOG Ab were observed in many patients, and these patients could not develop high affinity MOG Ab. Parallel to findings in an autoimmune lupus murine model [22], our results may suggest that limited changes to antibody affinity occur across MOG-specific B cell clones despite some hallmarks of affinity maturation, such as isotype-switching to IgG.

Limitations of the present study include the potential referral bias of the cohort as relapsing patients may be more likely to be referred for testing, and the unconfirmed disease onset among some patients of our longitudinal cohort in whom baseline samples may therefore not reflect the first acute episode of disease onset. We also observed fluctuating MOG Ab titers over time, but were unable to determine whether these changes were influenced by immunotherapy, which has been observed in previous reports [12, 25]. Furthermore, a single epitope was studied in this cohort. However, the MOG Ab response was largely dominated by Proline42 reactivity, and responses against additional epitopes comprise a smaller proportion of patients as reported in children [34]. Prospective data to assess the predictive value of antibody titre and epitope may be needed in the future.

The current study demonstrates the binding sensitivity of the human MOG Ab response, which sheds critical light on the importance of antigen conformation and highlights the caveats in the routine detection of human autoantibody. The characterisation of the human MOG Ab by affinity and epitope immunoreactivity provides a foundation for future pathogenic studies in animal models, B cells studies in human, and new avenues to improve patient diagnoses and management.

## Additional file

Additional file 1: **Figure S1.** Human native-MOG Ab is not of an IgM isotype. **Figure S2.** Assessment of MOG Ab titers in serum. **Figure S3.** Distribution of native-MOG Ab with age. **Figure S4.** Detection of human native-MOG Ab in fixed flow and biochip assays. **Figure S5.** P42 is an immunodominant epitope in paediatric and adult MOG Ab responses. **Figure S6.** High affinity Ab have stable immunoreactivity to P42 and do not correlate with native-MOG or fixed-MOG Ab titers. **Table S1.** Intra-assay variability of live and fixed flow assays. **Table S2.** Clinical characteristics of longitudinal native-MOG Ab seropositive patients. **Table S3.** Comparison of MOG Ab index in serum and CSF. **Table S4.** Clinical phenotypes of native-MOG Ab seropositive patients undetected in fixed flow and biochip assays. **Supplementary material 1.** Clinical vignettes of native-MOG Ab seropositive patients undetected in fixed flow and biochip assays. (DOCX 1186 kb)

## Data Availability

Correspondence and requests for data or materials should be addressed to FB. Plasmids transfer should be obtained through a Material Transfer Agreement.
