# Supplementary material for: Characterization of the human myelin oligodendrocyte glycoprotein antibody response in demyelination
Source: Acta Neuropathol Commun. 2019 Sep 3;7:145. doi: 10.1186/s40478-019-0786-3 (PMC6724269; doi:10.1186/s40478-019-0786-3)
Supplement: Supplementary file 1 — Figure S1. Human native-MOG Ab is not of an IgM isotype. Figure S2. Assessment of MOG Ab titers in serum. Figure S3. Distribution of native-MOG Ab with age. Figure S4. Detection of human native-MOG Ab in fixed flow and biochip assays. Figure S5. P42 is an immunodominant epitope in paediatric and adult MOG Ab responses. Figure S6. High affinity Ab have stable immunoreactivity to P42 and do not correlate with native-MOG or fixed-MOG Ab titers. Table S1. Intra-assay variability of live and fixed flow assays. Table S2. Clinical characteristics of longitudinal native-MOG Ab seropositive patients. Table S3. Comparison of MOG Ab index in serum and CSF. Table S4. Clinical phenotypes of native-MOG Ab seropositive patients undetected in fixed flow and biochip assays. Supplementary material 1. Clinical vignettes of native-MOG Ab seropositive patients undetected in fixed flow and biochip assays. (DOCX 1186 kb) [file 40478_2019_786_MOESM1_ESM.docx]

**Electronic Supplementary Material**

# CHARACTERIZATION OF THE HUMAN MYELIN OLIGODENDROCYTE GLYCOPROTEIN ANTIBODY RESPONSE IN DEMYELINATION

**F Tea et al.**

**
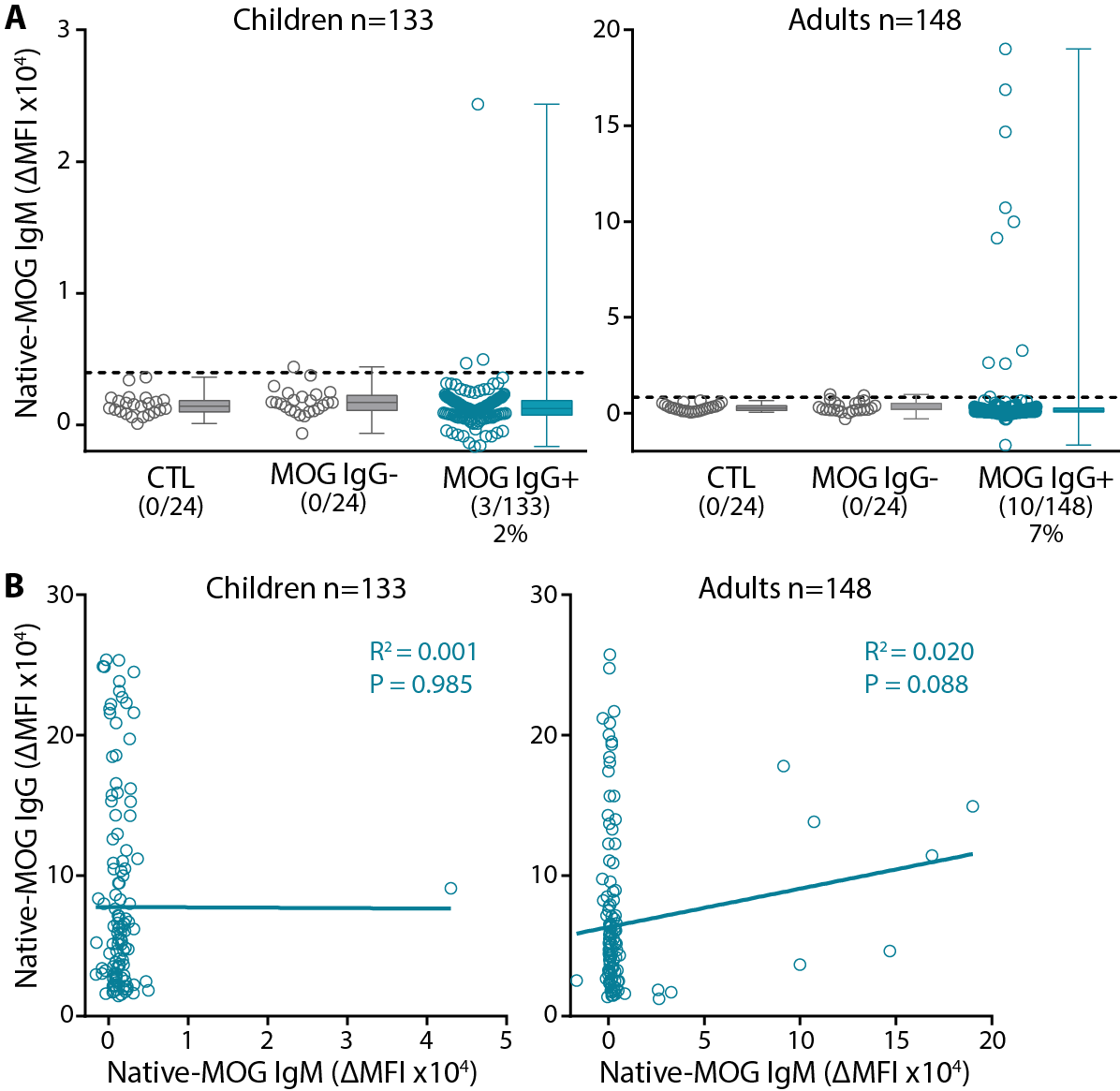
**

**Figure S1. Human native-MOG Ab is not of an IgM isotype. (A)** Only 3/133 (2.2%) paediatric and 10/148 (6.8%) adult patients harboured MOG IgM Ab by live flow assay. In the MOG IgG Ab- groups, one paediatric and 2 adult sera were positive for MOG IgM Ab. (**B)** There was no correlation between IgG (H+L) and IgM levels across all patients, and MOG IgM Ab+ patients exhibited a range of MOG IgG titres. CTL = control, IgM = immunoglobulin M, MFI = median fluorescence intensity, MOG IgG- = MOG antibody negative, MOG IgG+ = MOG antibody positive.

**
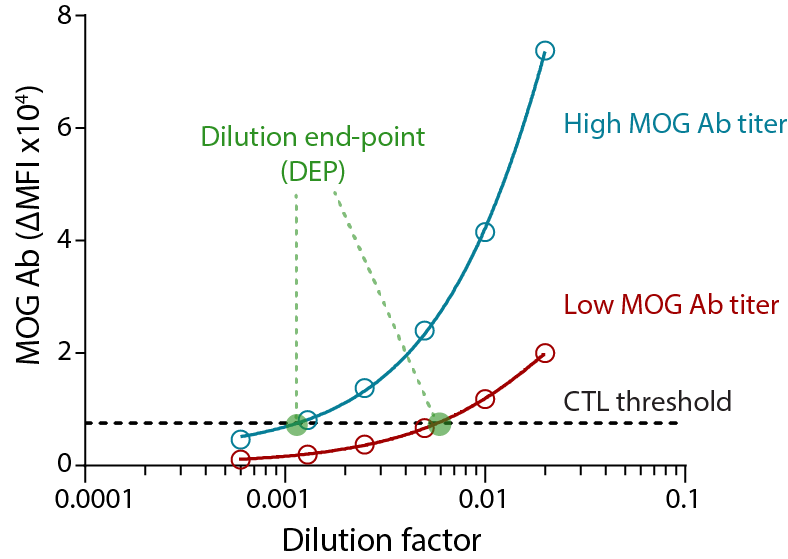
**

**Figure S2. Assessment of MOG Ab titers in serum.** By live flow assay, six serial dilutions (1:50 – 1:1600) were performed with patient sera (blue and red dots). Sigmoidal regression curves were fitted between ΔMFI and dilution values (blue and red line). Age-matched controls (24 children, 24 adults) at 1:50 were run in parallel to establish the control threshold (mean of age-matched controls + 3SD, black dotted line). The dilution-end-point (DEP, green) was interpolated at the control threshold. CTL = control, DEP = dilution end-point, MFI = median fluorescence intensity, MOG Ab = MOG antibody


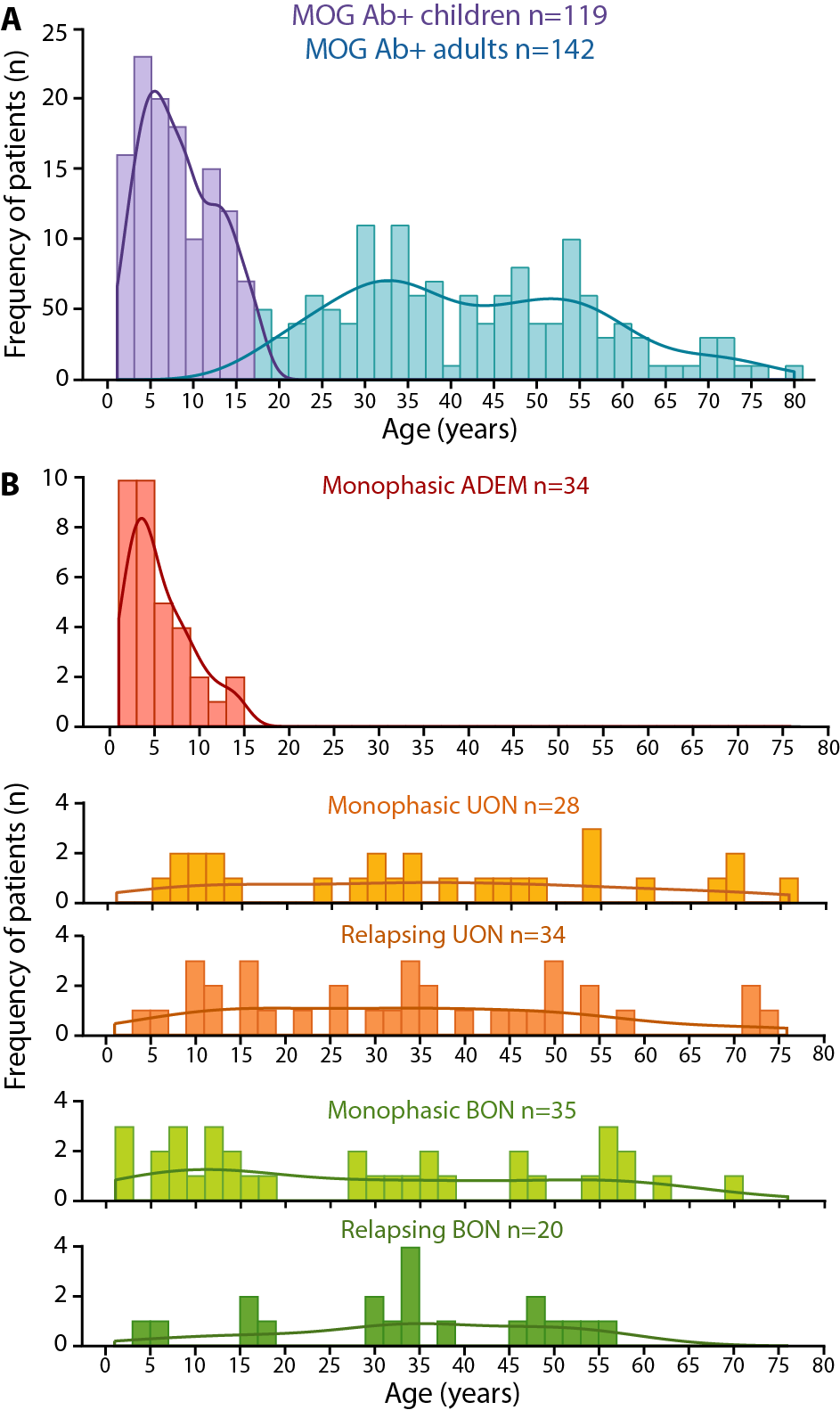


**Figure S3. Distribution of native-MOG Ab with age**. **(A)** Distribution of native-MOG Ab with age across children (purple) and adults (blue). **(B)** Distribution of age among MOG Ab-associated phenotypes. ADEM (red) was observed in children, whereas UON (orange) and BON (green) were spread over a wide age distribution. Age was unknown in 20 children and 6 adults. ADEM = Acute disseminated encephalomyelitis; BON = Bilateral optic neuritis; UON = Unilateral optic neuritis.


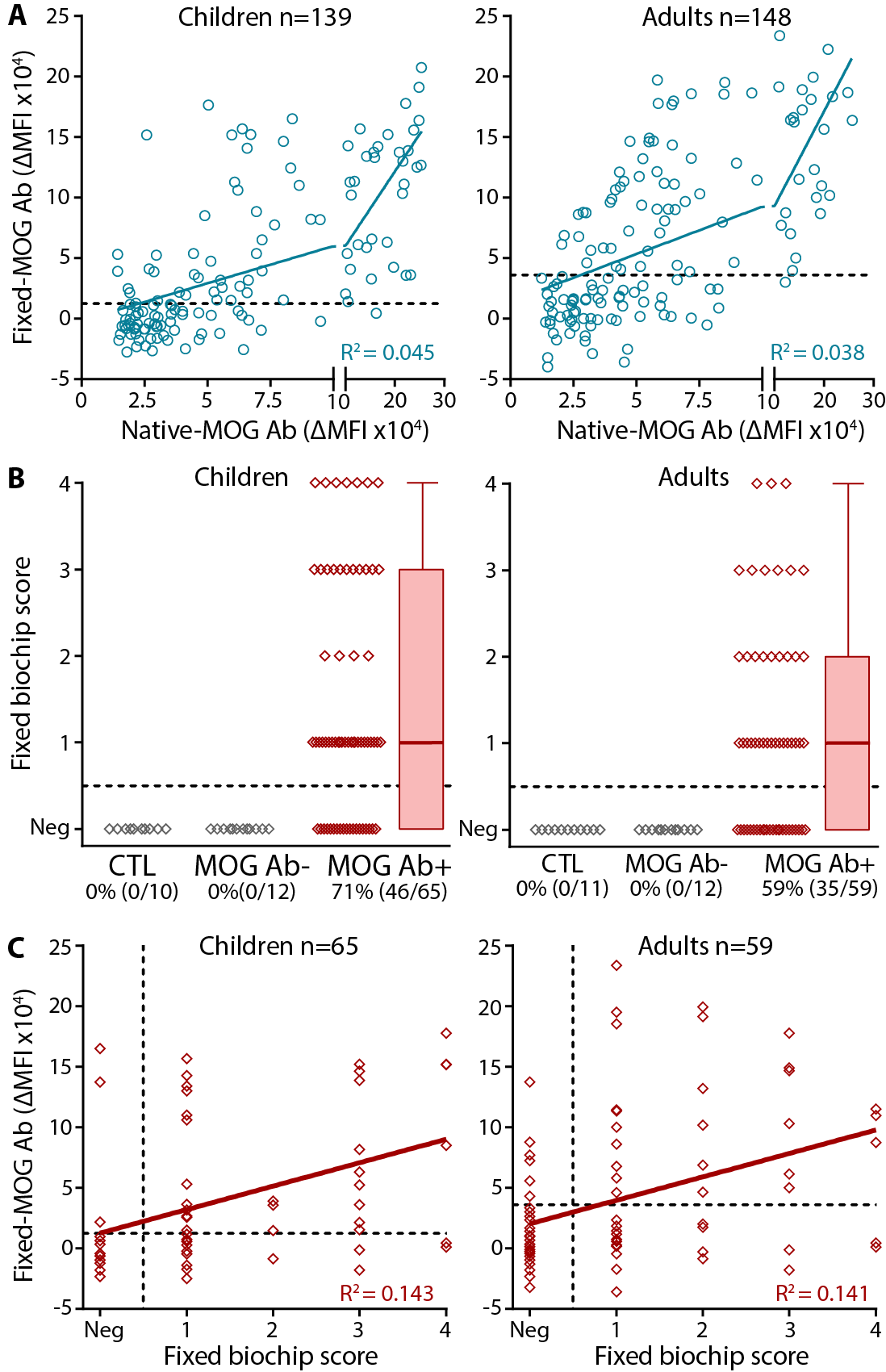


**Figure S4. Detection of human native-MOG Ab in fixed flow and biochip assays.** **(A)** Native-MOG Ab titers were poorly correlated with fixed-MOG Ab titers in children (R^2^=0.045, P<0.0001) and adults (R^2^=0.038, P<0.0001). **(B)** Seropositivity by fixed biochip assay was determined in 65 native-MOG Ab+ children (red) and 59 adults (red) with demyelinating disorders. Only 46/65 (71%) native-MOG Ab+ children and 35/59 (59%) adult sera were able to bind fixed-MOG in the biochip assay. Fixed-MOG Ab were not detected in all paediatric (n=10) and adult (n=11) control sera, and all native-MOG Ab- paediatric (n=12) and adult (n=12) sera (grey). **(C)** Fixed biochip score determined by independent examiners did not correlate with fixed-MOG Ab titers quantified in the fixed flow assay (R^2^=0.143, P>0.05, children; R^2^=0.141, P>0.05, adults). Dotted lines represent the positive threshold in each respective assay. Ab- = antibody negative, Ab+ = antibody positive, CTL = controls, MFI = median fluorescence intensity, Neg = MOG Ab seronegative by fixed biochip assay.


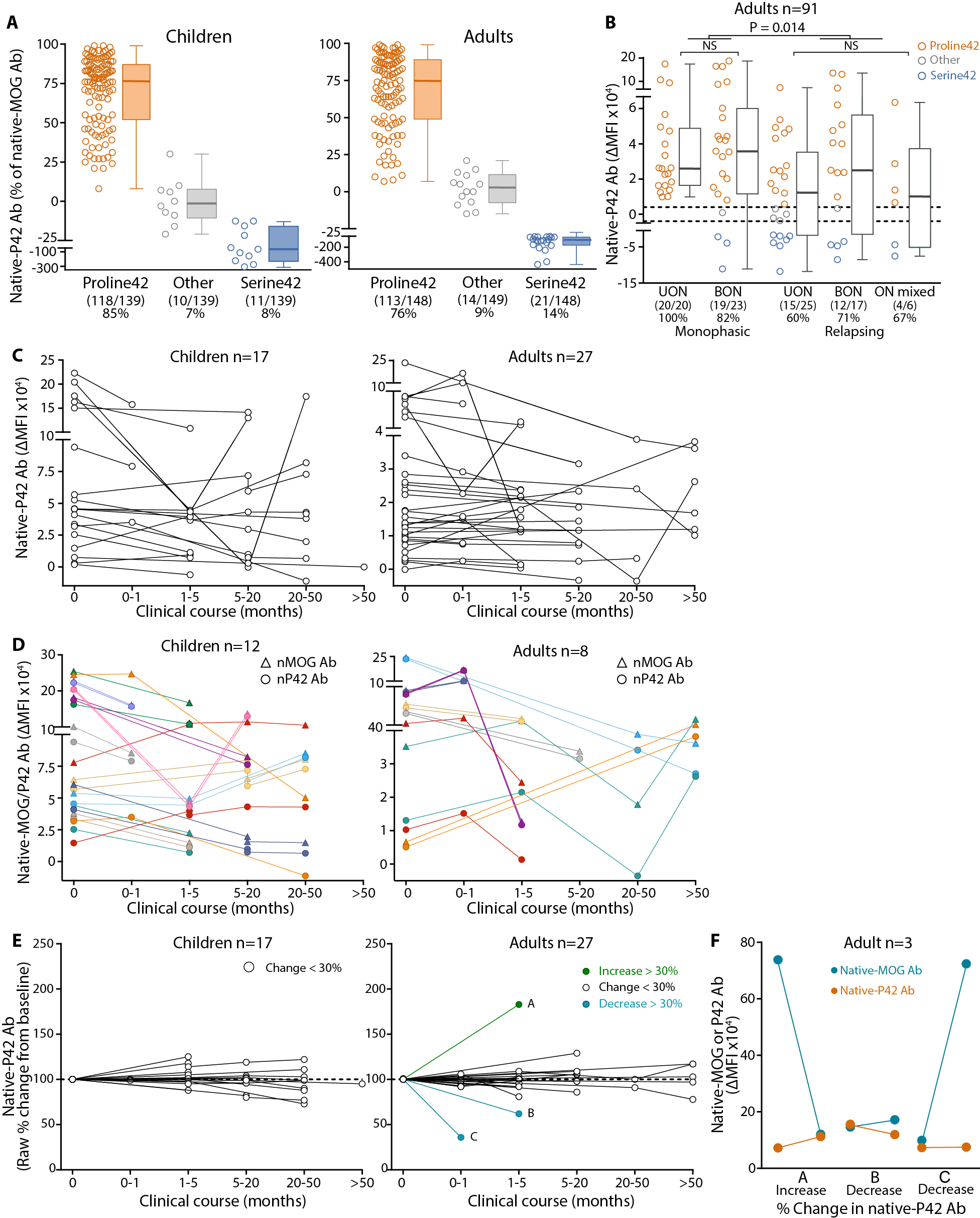


**Figure S5. P42 is an immunodominant epitope in paediatric and adult MOG Ab responses**. **(A)** With patients categorized using a control reference range**,** P42 Ab made up 75-77% of total native-MOG Ab response in children and adults (orange). **(B)** Adults with a relapsing ON course (UON, BON, or ON mixed) had lower immunoreactivity to P42 than adults with a monophasic disease course (P=0.014). Dotted lines indicate the control reference range determined by age-matched controls (n=24 children, n=24 adults). (**C and D**) Native-P42 Ab titers were analysed over time in children (n=17) and adults (n=27). Native-P42 Ab titers (circles) paralleled native-MOG Ab titers (triangles), even in patients with significant fluctuations in individual patients as represented by different colors (n=12 children, n=8 adults). **(E)** Compared to baseline sample, P42 Ab titers represented by the dilution end-point (DEP) remained stable (<30% change) in all children (n=17) and 24/27 adults. One adult had increased titers (Patient A, green), and two adults had decreased titers of P42 Ab (Patients B and C, blue). Dotted lines represent P42 Ab titer (100%) at the baseline. **(F)** Raw native-MOG and native-P42 Ab titers shown for Patients A, B, and C. Titers of native-P42 Ab remain relatively stable, however, native-MOG Ab titers change significantly. Ab = antibody, BON = bilateral optic neuritis, DEP = dilution end-point, MFI = median fluorescence intensity, native-P42 Ab = native Proline42 binding MOG Ab, ON mixed = combination of BON and UON, P42 = Proline 42, S42 = Serine 42, UON = unilateral optic neuritis.

**
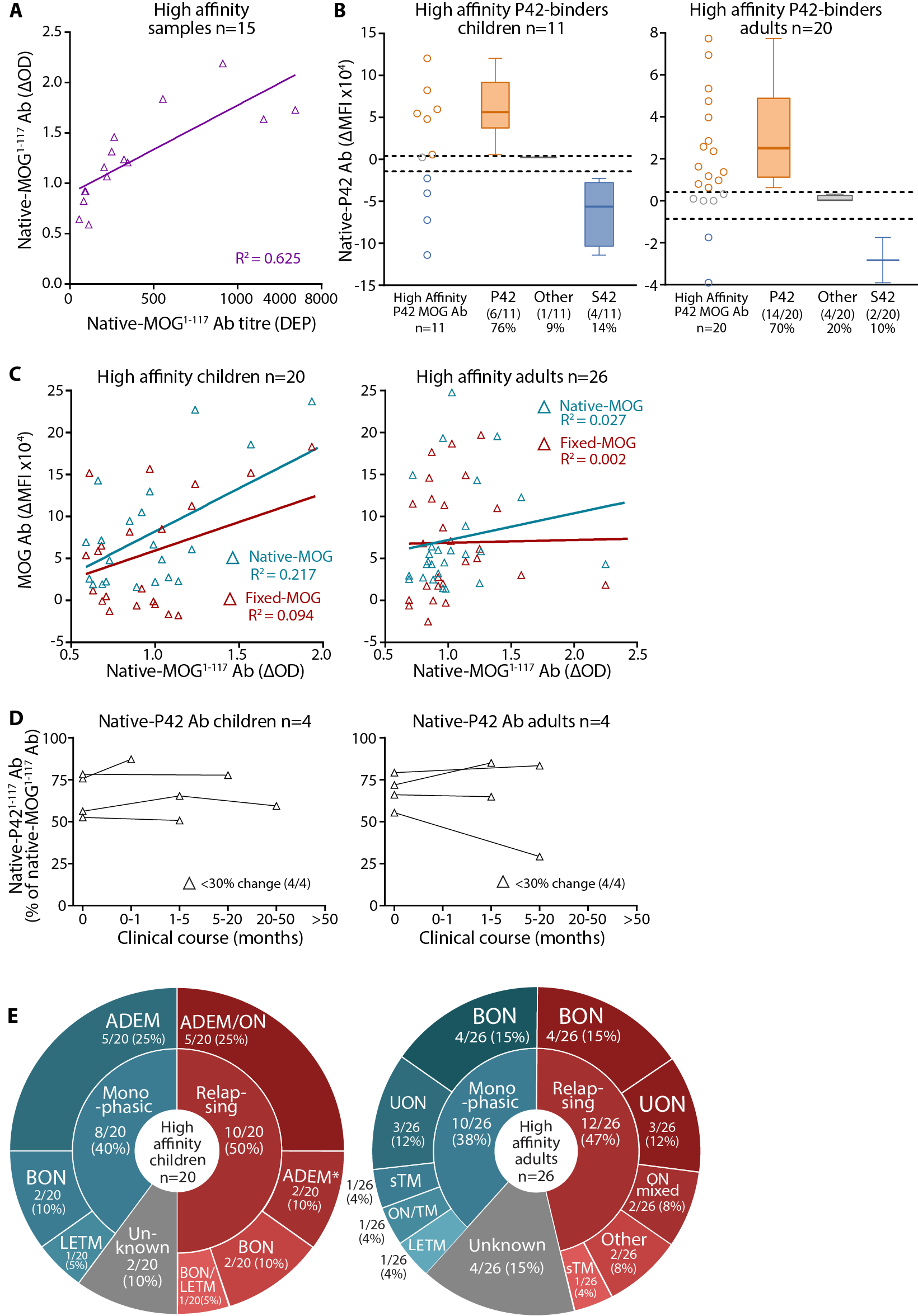
**

**Figure S6. High affinity Ab have stable immunoreactivity to P42 and do not correlate with native-MOG or fixed-MOG Ab titers. (A)** There was a correlation between the ΔOD of serum diluted at 1:50 and native-MOG^1-117^ Ab titers represented by dilution end-point (DEP) in children with high affinity Ab (P=0.0004, R^2^=0.625). **(B)** Among the high affinity P42^1-117^ binders (n=11 children, n=20 adults), most of these patients bound native-P42 Ab (6/11 children, 55%; 14/20 adults, 70%). Dotted line represents the control reference range determined by age-matched controls (n=24 children, n=24 adults). Number and percentage of patients in each epitope category are shown, and one representative out of three independent experiments is shown. **(C)** Native-MOG^1-117^ Ab titer was not associated with native-MOG (blue) or fixed-MOG (red) Ab titers in children (left) and adults (right). **(D)** High affinity Ab immunoreactivity toward P42 did not change (<30% change) regardless of baseline P42 Ab titers in all four paediatric (left) and all four adult (right) sera. **(E)** Distribution of high affinity MOG Ab among paediatric (left) and adult (right) clinical phenotypes**. ***Relapsing ADEM is multiphasic ADEM according to [[29](#_ENREF_29)]. ADEM = acute disseminated encephalomyelitis, BON = bilateral optic neuritis, DEP = dilution end point, LETM = longitudinally extensive transverse myelitis, native-P42 Ab = native-MOG antibodies binding to Proline42, OD = optical density, ON mixed = combination of BON and UON, ON/TM = simultaneous ON and transverse myelititis, relapsing ADEM = multiphasic ADEM, sTM = short transverse myelitis, UON = Unilateral optic neuritis. DEP = dilution end-point, Native-MOG^1-117^ Ab = high affinity MOG Ab binding to extracellular MOG, OD = optical density, P42^1-117^ Ab = MOG Ab binding to extracellular Proline42.

**Table S1.** Intra-assay variability of live and fixed flow assays.

|  | | **Live flow assay** | | | **Fixed flow assay** | | |
| --- | --- | --- | --- | --- | --- | --- | --- |
|  |  | CTL | MOG Ab- | MOG Ab+ | CTL | MOG Ab- | MOG Ab+ |
| Children *N* |  | 24 | 24 | 139 | 24 | 24 | 139 |
| Times MOG Ab+ of three experiments  *(% total)* | 0/3 | 24 (100) | 24 (100) | 0 | 24 (100) | 24 (100) | 38 (27) |
|  | 1/3 | 0 | 0 | 0 | 0 | 0 | 23 (17) |
|  | 2/3 | 0 | 0 | 8 (6) | 0 | 0 | 25(18) |
|  | 3/3 | 0 | 0 | 131 (94) | 0 | 0 | 53 (38) |
| Adults *N* |  | 24 | 24 | 148 | 24 | 24 | 148 |
| Times MOG Ab+ of three experiments  *(% total)* | 0/3 | 24 (100) | 23 (96) | 0 | 23 (96) | 16 (67) | 38 (26) |
|  | 1/3 | 0 | 1 (4) | 0 | 1 (4) | 8 (33) | 33 (22) |
|  | 2/3 | 0 | 0 | 6 (4) | 0 | 0 | 25 (17) |
|  | 3/3 | 0 | 0 | 142 (96) | 0 | 0 | 52 (35) |

CTL = Control patients, MOG Ab- = MOG antibody seronegative patients, MOG Ab+ = MOG antibody seropositive patients.

**Table S2.** Clinical characteristics of longitudinal native-MOG Ab seropositive patients.

|  | **Children** | | | | **Adults** | | | |
| --- | --- | --- | --- | --- | --- | --- | --- | --- |
|  | All longitudinal patients  *N (%)* | Disease duration  *Median* *months* (IQR, min-max)*^,^** | Persistent native-MOG Ab  *N (%) **** | Disease duration *Median* *months*  (IQR, min-max)*^,^** | All longitudinal patients  *N (%)* | Disease duration *Median* *months* (IQR, min-max)*^,^** | Persistent native-MOG Ab  *N (%)**** | Disease duration *Median* *months* (IQR, min-max)*^,^** |
| **Longitudinal patients** | 19 (all) | 17.6 (3.6-28.9) | 15 (all) | 19.3 (6.1-33.1) | 32 (all) | 4.1 (1.7-13.9) | 20 (all) | 10.6 (4.5-61.3) |
| Total samples collected | 54 |  | 25 |  | 76 |  | 52 |  |
| Monophasic course | 3 (16 of all) | 19.3 (17.6-56.7) | 3 (20 of all) | 19.3 (17.6-56.7) | 8 (25 of all) | 4.8 (1.1-12.1) | 5 (25 of all) | 6.5 (4.8-45.6) |
| Monophasic BON | 1 (33) | 17.6 (-) | 1 (33) | 17.6 (-) | 4 (50) | 3.8 (1-12.1) | 2 (40) | 10.2 (6.5-13.9)* |
| Monophasic UON | - | - | - | - | 1 (13) | 5.7 (-) | 1 (20) | 5.7 (-) |
| Monophasic ADEM | 2 (67) | 38.0 (19.3-56.7)* | 2 (67) | 38.0 (19.3-56.7)* | - | - | - | - |
| Monophasic LETM | - | - | - | - | 2 (25) | 2.5 (1.1-3.9)* | 1 (20) | 3.9 (-) |
| Monophasic LETM/Brainstem | - | - | - | - | 1 (13) | 77.2 (-) | 1 (20) | 77.2 (-) |
| Relapsing course | 16 (84 of all) | 11.3 (2.1-26.8) | 12 (80 of all) | 19 (5.4-32) | 22 (69 of all) | 4.1 (2.3-43.8) | 14 (70 of all) | 11.5 (4.2-62.4) |
| Relapsing BON | 2 (13) | 3.1 (0.1-6.1)* | 1 (8) | 6.1 (-) | 9 (41) | 3.9 (2.6-60.5) | 6 (43) | 32.1 (3.7-62.4) |
| Relapsing UON | 3 (19) | 3.6 (1.6-16.4) | 3 (25) | 10.0 (3.6-16.4) | 8 (36) | 6.7 (1.9-64) | 5 (36) | 38.7 (6.7-90.9) |
| Relapsing ON mixed | - | - | - | - | 3 (14) | 8.7 (1.8-13.9) | 2 (14) | 11.3 (8.7-13.9)* |
| Relapsing ON/LETM | 3 (19) | 18.7 (0.2-19.3) | 2 (17) | 19.0 (18.7-19.3)* | 1 (5) | 3.5 (-) | 1 (7) | 3.5 (-) |
| Relapsing ADEM**** | 3 (19) | 20.6 (5.1-37.1) | 3 (25) | 20.6 (5.1-37.1) | 1 (5) | 1.1 (-) | - | - |
| Relapsing ADEM/ON | 3 (19) | 28.9 (0.2-35.9) | 2 (8) | 32.4 (28.9-35.9)* | - | - | - | - |
| Relapsing ADEM/LETM | 1 (6) | 33.1 (-) | 1 (8) | 33.1 (-) | - | - | - | - |
| Relapsing ADEM/ON/LETM | 1 (6) | 4.8 (-) | 1 (8) | 4.8 (-) | - | - | - | - |
| Unknown | - | - | - |  | 2 (6 of total) | 6.6 (1.0-12.1)* | 1 (5) | 12.1 (-) |

*Median (min-max) if N samples < 3. **IQR=interquartile range if N samples ≥ 3, ***Persistent MOG Ab when patient maintained positive serostatus for more than three months. ****Relapsing ADEM is multiphasic ADEM according to [[29](#_ENREF_29)].

**Table S3.** Comparison of MOG Ab index in serum and CSF.

|  | Patient | CSF MOG Ab (1:1, ΔMFI x10^3^) | Serum MOG Ab (1:1, ΔMFI x 10^-3^) | QMOG  (x 10^-3^)* | QIgG  (mg/dL)** | MOG Ab index  *** | CSF MOG Ab for MOG Ab index > 4**** | Clinical phenotype |
| --- | --- | --- | --- | --- | --- | --- | --- | --- |
| Children MOG Ab+ in CSF and serum | 1 | 4.0 | 1129 | 3.5 | 2.0 | 1.81 | 8.8 | Monophasic ADEM |
|  | 1a***** | 4.0 | 1043 | 3.8 | 2.2 | 1.71 | 9.4 | Monophasic ADEM |
|  | 2 | 2.3 | 2566 | 0.9 | 1.4 | 0.66 | 14.0 | Monophasic LETM |
|  | 3 | 3.2 | 1143 | 2.8 | 4.6 | 0.62 | 20.8 | Monophasic ADEM |
|  | 4 | 11.5 | 5463 | 2.1 | 4.0 | 0.53 | 86.3 | Monophasic BON |
|  | 5 | 18.9 | 5265 | 3.6 | 7.3 | 0.49 | 154.5 | Relapsing BON/LETM |
|  | 6 | 2.1 | 3214 | 0.7 | 1.6 | 0.41 | 20.7 | Monophasic BON |
|  | 7 | 0.7 | 1548 | 0.4 | 1.2 | 0.36 | 7.5 | Relapsing BON/LETM |
|  | 8 | 1.1 | 881 | 1.2 | 5.3 | 0.23 | 18.7 | Monophasic ADEM |
|  | 9 | 1.3 | 2387 | 0.5 | 2.3 | 0.22 | 22.4 | Monophasic ADEM |
|  | 9a | 11.4 | 12113 | 0.9 | 4.5 | 0.21 | 219.0 | Monophasic LETM |
|  | 10 | 4.8 | 11361 | 0.4 | 2.4 | 0.18 | 108.9 | Relapsing ADEM |
| Adults MOG Ab+ in CSF and serum | 1 | 72.8 | 538 | 135.3 | 8.5 | 15.88 | 18.3 | Relapsing UON |
|  | 2 | 25.7 | 1447 | 17.8 | 1.7 | 10.35 | 9.9 | Relapsing UON |
|  | 3 | 5.1 | 1472 | 3.5 | 3.2 | 1.08 | 18.8 | Relapsing UON |
|  | 4 | 6.7 | 3482 | 1.9 | 2.4 | 0.81 | 32.8 | Monophasic LETM |
|  | 5 | 1.8 | 1017 | 1.8 | 3.6 | 0.50 | 14.5 | Monophasic ADEM |
|  | 6 | 4.8 | 1484 | 3.3 | 7.1 | 0.46 | 42.3 | Monophasic BON |
|  | 7 | 1.9 | 2024 | 0.9 | 2.5 | 0.38 | 20.1 | Monophasic UON |
|  | 8 | 2.0 | 3807 | 0.5 | 2.3 | 0.23 | 34.3 | Relapsing BON |
|  | 8a | 5.3 | 5434 | 1.0 | 4.9 | 0.20 | 107.4 | Relapsing BON |
|  | 9 | 0.3 | 970 | 0.3 | 13.5 | 0.03 | 52.5 | Relapsing BON |

*QMOG = CSF MOG Ab ΔMFI / Serum MOG Ab ΔMFI, **QIgG = Total CSF IgG / Total serum IgG, ***MOG Ab index = QMOG / QIgG. **** MOG Ab index >4 indicates intrathecal MOG Ab production as described in [26, 27]. *****“a” indicates serial CSF from the same patient. CSF = cerebrospinal fluid, MOG Ab+ = native-MOG Ab seropositive patients, QIgG = Quotient IgG, QMOG = Quotient MOG Ab. Median total CSF IgG in children = 2.3 (IQR 1.9-3.9) mg/dL, adults = 2.6 (IQR 1.6-6.6) mg/dL. Median total Serum IgG in children = 960 (IQR 658-1153) mg/dL, adults = 815 (IQR 651-920) mg/dL. ADEM = acute disseminated encephalomyelitis, BON = bilateral optic neuritis, CIS = clinically isolated syndrome, LETM = longitudinally extensive transverse myelitis, MOG Ab- = MOG antibody seronegative patients, MOG Ab+ = MOG antibody seropositive patients, ON mixed = combination of BON and UON, ON/TM = simultaneous ON and transverse myelitis, relapsing ADEM = multiphasic ADEM, UON = Unilateral optic neuritis.

**Table S4.** Clinical phenotypes of native-MOG Ab seropositive patients undetected in fixed flow and biochip assays.

|  | **Children** | | | | | | | **Adults** | | | | | | | |
| --- | --- | --- | --- | --- | --- | --- | --- | --- | --- | --- | --- | --- | --- | --- | --- |
|  | **Fixed flow assay** | | | **Fixed biochip assay** | | | Responsive | **Fixed flow assay** | | **Fixed biochip assay** | | | | | Responsive to therapy  *N (% total)** |
|  | MOG Ab+ | MOG Ab- | MOG Ab+ | | MOG Ab- | to therapy | | MOG Ab+ | MOG Ab- | | | MOG Ab+ | | MOG Ab-  *N (% total)* |  |
|  | *N (% total)* | *N (% total)* | *N (% total)* | | *N (% total)* | *N (% total)** | | *N (% total)* | *N (% total)* | | | *N (% total)* | |  |  |
| All phenotypes (all) | 78 (all) | 61 (all) | 46 (all) | | 19 (all) | 19 (all) | | 79 (all) | 69 (all) | | 35 (all) | | 24 (all) | | 28 (all) |
| Monophasic course | 43 (55) | 36 (59) | 24 (52) | | 12 (63) | 8 (42) | | 32 (41) | 25 (36) | | 14 (40) | | 5 (21) | | 10 (36) |
| Relapsing course | 23 (30) | 16 (26) | 16 (35) | | 6 (32) | 11 (58) | | 33 (42) | 28 (41) | | 11 (31) | | 13 (54) | | 15 (54) |
| Unknown | 12 (15) | 9 (15) | 6 (13) | | 1 (5) | - | | 14 (17) | 16 (23) | | 10 (29) | | 6 (25) | | 3 (11) |
| ON | 21 (27 of all) | 17 (28 of all) | 12 (26 of all) | | 5 (26 of all) | 7 (37 of all) | | 50 (72 of all) | 41 (59 of all) | | 19 (54 of all) | | 14 (58 of all) | | 17 (61 of all) |
| Monophasic ON | 12 (57) | 10 (59) | 9 (75) | | 1 (20) | 2 (29) | | 23 (46) | 20 (49) | | 12 (63) | | 5 (36) | | 7 (41) |
| Relapsing ON | 9 (43) | 7 (41) | 3 (25) | | 4 (80) | 5 (71) | | 27 (54) | 21 (51) | | 7 (37) | | 9 (64) | | 10 (59) |
| BON | 10 (47 of ON) | 8 (47 of ON) | 8 (66 of ON) | | 1 (2 of ON) | 3 (43 of ON) | | 27 (54 of ON) | 13 (32 of ON) | | 10 (53 of ON) | | 4 (29 of ON) | | 7 (41 of ON) |
| Monophasic BON | 8 (80) | 6 (75) | 7 (88) | | - | 1 (33) | | 15 (56) | 8 (62) | | 6 (60) | | 2 (0.5) | | 4 (57) |
| Relapsing BON | 2 (20) | 2 (25) | 1 (12) | | 1 (100) | 2 (67) | | 12 (44) | 5 (38) | | 4 (40) | | 2 (0.5) | | 3 (43) |
| UON | 9 (43 of ON) | 8 (47 of ON) | 2 (17 of ON) | | 4 (80 of ON) | 3 (43 of ON) | | 20 (40 of ON) | 25 (61 of ON) | | 9 (47 of ON) | | 8 (57 of ON) | | 7 (41 of ON) |
| Monophasic UON | 4 (44) | 4 (50) | 2 (100) | | 1 (25) | 1 (33) | | 8 (40) | 12 (48) | | 6 (67) | | 3 (38) | | 4 (57) |
| Relapsing UON | 5 (56) | 4 (50) | - | | 3 (75) | 2 (67) | | 12 (60) | 13 (52) | | 3 (33) | | 5 (63) | | 3 (43) |
| Relapsing ON mixed | 2 (10 of ON) | 1 (6 of ON) | 2 (17 of ON) | | - | 1 (14 of ON) | | 3 (6 of ON) | 3 (7 of ON) | | - | | 2 (14 of ON) | | 3 (18 of ON) |
| ADEM | 31 (40 of all) | 22 (36 of all) | 19 (41 of all) | | 9 (47 of all) | 5 (26 of all) | | - | 1 (1 of all) | | - | | 1 (4 of all) | | - |
| Monophasic ADEM | 25 (80) | 17 (77) | 14 (74) | | 7 (78) | 3 (60) | | - | - | | - | | - | | - |
| Relapsing ADEM** | 6 (20) | 5 (23) | 5 (26) | | 2 (22) | 2 (40) | | - | 1 (1) | | - | | 1 (1) | | - |
| ON/TM | - | - | - | | - | - | | 4 (6 of all) | 5 (7 of all) | | 4 (12 of all) | | - | | 3 (11 of all) |
| Monophasic ON/TM | - | - | - | | - | - | | 1 (25) | 2 | | 1 (25) | | - | | 1 (33) |
| Relapsing ON/TM | - | - | - | | - | - | | 3 (75) | 3 (100) | | 3 (75) | | - | | 2 (67) |
| LETM | 5 (6 of all) | 5 (7 of all) | 1 (2 of all) | | 3 (16 of all) | 1 (5 of all) | | 6 (9 of all) | 2 (3 of all) | | 1 (3 of all) | | 1 (5 of all) | | 2 (7 of all) |
| Monophasic LETM | 5 (100) | 5 (100) | 1 (100) | | 3 (100) | 1 (100) | | 6 (100) | 1 (50) | | 1 (100) | | - | | 1 (50) |
| Relapsing LETM | - | - |  | | - |  | | - | 1 (50) | | - | | 1 (100) | | 1 (50) |
| Relapsing ON/ADEM | 6 (8 of all) | 4 (7 of all) | 5 (11 of all) | | - | 3 (16 of all) | | - | - | | - | | - | | - |
| Monophasic CIS | - | 3 (3 of all) | - | | - | 1 (5 of all) | | - | - | | - | | - | | - |
| Other*** | 3 (4 of all) | 1 (3 of all) | 2 (4 of all) | | - | 1 (5 of all) | | 4 (6 of all) | 3 (4 of all) | | 4 (11 of all) | | 1 (4 of all) | | 2 (7 of all) |
| Phenotypes n<3**** | 3 (4 of all) | 3 (2 of all) | 1 (2 of all) | | 1 (5 of all) | 1 (5 of all) | | 5 (7 of all) | 4 (6 of all) | | 1 (3 of all) | | 2 (8 of all) | | 3 (11 of all) |
| Unknown Phenotype | 9 (12 of all) | 7 (11 of all) | 6 (12 of all) | | 1 (5 of all) | - | | 10 (14 of all) | 13 (19 of all) | | 6 (17 of all) | | 5 (21 of all) | | 1 (4 of all) |

*Patients detected negative by fixed flow or fixed biochip assays responsive to immunotherapies including; Intravenous methylprednisolone and/or oral prednisone (17 children, 19 adults); Azathioprine (4 adults); Intravenous immunoglobulin (2 children, 2 adults), Rituximab (1 child, 4 adults); Plasmapheresis (1 child, 1 adults); Plasma exchange (2 adults); Mycophenolate (1 adult); Copaxone (1 adult); 1 child and 6 adults were treated with a combination of >1 immunotherapies. **Relapsing ADEM is multiphasic ADEM according to [30]. ***Other: uncommon and atypical MOG Ab-associated phenotypes, including seizures, cerebellar, brainstem, brainstem and headache and fatigue, acute psychosis, optic perineuritis. ****Phenotypes with less than three patients; children, monophasic LETM/UON (1), monophasic short TM (1), relapsing BON/LETM (2), relapsing BON/LETM/ADEM (1), relapsing LETM/UON (1); adults, monophasic short TM (2), monophasic LETM/ADEM (1), monophasic LETM/Brainstem (1), relapsing short TM (2), relapsing TM mixed (1), relapsing BON/LETM/ADEM (1), relapsing ON//Brainstem/short TM (1). ADEM = acute disseminated encephalomyelitis, BON = bilateral optic neuritis, CIS = clinically isolated syndrome, LETM = longitudinally extensive transverse myelitis, MOG Ab- = MOG antibody seronegative patients, MOG Ab+ = MOG antibody seropositive patients, ON mixed = combination of BON and UON, ON/TM = simultaneous ON and transverse myelitis, relapsing ADEM = multiphasic ADEM, UON = Unilateral optic neuritis.

**Supplementary Material 1: Clinical vignettes of native-MOG Ab seropositive patients undetected in fixed flow and biochip assays.**

Child 1

A previously well 3 year old girl presented with a cluster of focal seizures but no other neurological symptoms or signs. EEG showed left centro-temporal slowing and epileptic discharges CSF showed no pleocytosis but elevated neopterin. MRI revealed a small inflammatory lesion in the contralateral white matter, which resolved on repeat MRI one week later. She was treated with carbamazepine and she had no recurrence of seizures. 8 months later she presented with encephalopathy, gait disturbance and MRI features of ADEM. Intravenous methylprednisolone (IVMP) then oral corticosteroids were used for 2 months, but 2 months after steroid cessation there was a further ADEM event. She was started on MMF and had a 6 month prednisolone taper, but relapsed with a further ADEM event 6 weeks after ceasing prednisolone. She has been relapse-free for the last 2 years on MMF 30mg/kg/day, plus 30mg of prednisolone twice weekly, and the prednisolone is being tapered by 5mg/dose every 6 months. Her MRI is now normal.

Diagnosis: MOG Ab-associated relapsing ADEM (multiphasic ADEM).

Child 2

A previously well 13 year old presented with a focal seizure and Todd’s paresis which resolved in 24 hours. Ten days later she developed headache and CSF pressure was 60 cm of water demonstrating raised intracranial pressure, with 150 white cells suggesting an infectious or inflammatory aetiology. Antibiotics were started but one week later she developed ataxia and drowsiness and MRI brain showed extensive inflammatory subcortical lesions, thalamic lesions and longitudinally extensive inflammatory lesion of the spinal cord. Intravenous methylprednisolone was given for 3 days followed by a 6 week taper of oral prednisolone and she had a complete recovery with normal MRI brain and spine at 3 month follow-up and 5 years of follow-up documented no relapse.

Diagnosis: MOG Ab-associated monophasic ADEM.

Child 3

A previously well 14 year old presented with acute onset gait disturbance, urinary retention and MRI spine showed inflammation from the cervical spine to the conus (complete transverse myelitis) with a normal MRI brain. Intravenous methylprednisolone for 3 days then oral prednisolone for 6 weeks was associated with a complete functional motor recovery within 3 months, but residual urinary voiding issues that required intermittent catheterization for the next 2 years. MRI spine repeat was normal, and after 5 years follow-up she had no further events and her urinary symptoms fully resolved.

Diagnosis: MOG Ab-associated monophasic TM.

Child 4

A previously well 14 year old presented with acute onset confusion, drowsiness and speech disturbance. MRI showed diffuse inflammatory lesions in the cortical grey matter and associated subcortical white matter. CSF showed mild pleocytosis with 3 polymorphs and 5 lymphocytes and negative oligoclonal bands and viral testing. Within 2 weeks she complained of left side visual loss and had clinical and radiological left optic neuritis. She was treated with intravenous methylprednisolone and 6 weeks of oral prednisolone and made a complete clinical recovery over the next one month. She has had no further clinical events for 2 years of follow-up.

Diagnosis: MOG Ab-associated monophasic ADEM.

Child 5

A previously well 4 year old boy presented with acute onset encephalopathy, confusion and bilateral visual loss. MRI showed extensive diffuse subcortical white matter lesions and optic nerve enhancement. CSF showed 20 lymphocytes and no oligoclonal bands. Intravenous methylprednisolone was given for 3 days then a 6 week oral prednisolone taper and he made a complete recovery and has had no relapses in 18 months of follow-up and repeat MRI 9 months later showed partial resolution of previous lesions and no new lesions.

Diagnosis: MOG Ab-associated monophasic ADEM.

Child 6

A previously well 10 year old presented with bilateral loss of vision, MRI consistent with bilateral optic nerve enhancement but normal MRI brain. CSF showed 3 monocytes and mirrored oligoclonal bands. She was treated with intravenous methylprednisolone for 3 days then oral prednisolone for 6 weeks taper. She made a complete functional visual recovery but has minor optic nerve pallor on fundoscopy. She has had no recurrence in 3 years of follow-up.

Diagnosis: MOG Ab-associated monophasic BON.

Adult 1

A 38 year old Caucasian female with a past history of migraines and preeclampsia in a previous pregnancy, presented with unilateral left-sided visual loss with no light perception, nausea, vomiting, truncal paraesthesia, urinary dysfunction, and saddle anaesthesia. MRI demonstrated left optic nerve swelling, enhancement, and hyperintensity; gadolinium enhancement of bilateral medullae with associated swelling and prominent involvement of the median raphe, bilateral cerebral peduncular involvement, and multilevel short segment thoracic cord hyperintensity (T6, T9, and T11). CSF examination revealed 14 white cells, normal protein, and no oligoclonal bands. She was treated immediately on presentation with three days of intravenous methylprednisolone (1g/day), followed by 50 mg daily of oral prednisone weaned over six weeks. She was acutely also treated with five courses of plasmapheresis. She had an excellent response to treatment with complete resolution of visual loss and only mild residual paraesthesia. However, two weeks post-cessation of oral steroids, she re-presented with unilateral right-sided visual loss and associated right-sided optic disc swelling. MRI demonstrated resolution of previous lesions and no new lesions. She was retreated with three days of intravenous methylprednisolone with complete restoration of vision, and restarted on oral prednisone as well as being commenced on azathioprine.

Diagnosis: MOG Ab-associated relapsing ON mixed (ON, TM, brainstem).

Adult 2

A 28 year old Tongan female presented with one week of bilateral pain with eye movement and reduced vision bilaterally (more prominent on the left), three weeks after a vaccination. She had bilateral optic disc swelling on examination, and MRI confirmed longitudinal left optic nerve swelling, hyperintensity, and diffuse mild enhancement with some perineural prominence and no demyelinating brain lesions. She was treated with three days of intravenous methylprednisolone (1g/day) with resolution of visual acuity within 48 hours of commencement. CSF examination revealed 30 monocytes in the presence of significantly blood-stained sample, normal protein, and negative oligoclonal bands. 18 months later, she developed right eye pain and reduced colour vision, with MRI evidence of right optic nerve enhancement and hyperintensity. She once again received three days of intravenous methylprednisolone with resolution of all symptoms within 24 hours of commencement. CSF examination at this time revealed four polymorphonuclear cells, normal protein, and negative oligoclonal bands.

Diagnosis: MOG Ab-associated relapsing ON mixed (BON/UON).

Adult 3

A 45 year old Sri Lankan female with no significant past history presented with a ten day history of right-sided visual loss (no light perception). She was treated with five days of intravenous methylprednisolone (1g/day) with some improvement in visual acuity over three weeks. MRI demonstrated two large left hemispheric frontal brain lesions with oedema and contrast enhancement which were atypical for multiple sclerosis, as well as three areas of periventricular hyperintensity. CSF examination did not have a lymphocytic pleocytosis. Protein was normal and oligoclonal bands were absent. Three months after this episode, she presented with bilateral visual loss which was once again treated with three days of intravenous methylprednisolone, with improvement in visual acuity. One month after this episode, she presented with deterioration in visual acuity bilaterally, and at this stage was retreated with three days of intravenous methylprednisolone. MRI demonstrated significant reduction in size of the two left frontal white matter changes. She was started on Avonex at this stage for a presumed diagnosis of atypical MS. Due to poor tolerability she was changed to glatiramer acetate one month later. Her diagnosis of MOG antibody seropositivity was made ten months following this episode, and she remains in remission at follow up seven years later.

Diagnosis: MOG Ab-associated relapsing BON.

Adult 3

An 18 year old Caucasian male with a past history of group A strep bacteraemia at one year of age, and Henoch-Schonlein purpura diagnosed at two years of age, presented with right-sided visual acuity loss which was confirmed on MRI with right optic nerve involvement and gadolinium enhancement. This episode resolved spontaneously, and was never treated. One year later, he had severe right-sided visual loss (no light perception), but was reluctant to present to medical attention or receive treatment. This episode was untreated, and his right visual acuity remained at counting fingers. Three years after this, he presented with severe visual acuity loss on the left at 6/60. At presentation, he received five days of intravenous methylprednisolone (1g/day), and was started on 25 mg od of oral prenisone, as well as being commenced on Avonex for a presumed diagnosis of multiple sclerosis. His visual acuity improved over two to three months on the left to 6/9. MRI brain and spine was normal. CSF examination revealed 6 mononuclear cells, normal protein, and intrathecal oligoclonal bands.Six months later, he had a fourth episode of left-sided visual acuity loss with a reduction in visual acuity to 6/24. At this stage, he was treated with five days of intravenous methylprednisolone (1g/day), commenced on 60 mg od of oral prednisone with slow tapering, had five plasma exchanges followed by a monthly exchange for ten months, and started on mycophenolate mofetil 1g bd. He was also treated with two courses of rituximab. Two years following this episode (and while on ongoing treatment with mycophenolate 1g bd and 5 mg od of prednisone, with rituximab retreatment 8 months prior), he represented with left-sided visual acuity loss. His prednisone dose was increased to 80 mg od with a slow wean, he received five further plasma exchanges followed by monthly plasmapheresis, and was retreated with rituximab. He remains under close follow up.

Diagnosis: MOG Ab-associated relapsing UON.

Adult 4

This 36 year old Indian female with a past history of migraine presented with two weeks of retro-orbital pain with eye movement, bilateral loss of visual acuity, and headaches. This occurred three weeks after a viral upper respiratory tract infection. Visual acuity at presentation was 6/60 OS and 6/36 OD with bilateral optic disc swelling. She received five days of intravenous methylprednisolone (1g/day) with resolution of vision to normal in the right eye with 48 hours, and to the left in 3 weeks. MRI brain demonstrated bilateral optic nerve hyperintensity, oedema, and subtle enhancement, as well as two small non specific lesions in the supratentorial white matter. CSF examination revealed no cells, an elevated protein level of 0.72 g/L, and negative oligoclonal bands. This patient was lost to follow-up.

Diagnosis: MOG Ab-associated monophasic BON.

Adult 5

This 44 year old Lebanese female with a past history of H pylori gastritis presented with a few weeks of a sensation of burning feet, bilateral upper limb sensory changes, and increasing lower limb incoordination and weakness. MRI demonstrated multilevel short segment spinal lesions (C3/T2/T3/T5). She was treated with three days of intravenous methylprednisolone (1g/day) with some mild improvement, but remained disabled. She was commenced on Avonex which was changed to glatiramer acetate due to poor tolerability of Avonex. Ten months later, she presented with increasing paraesthesia in bilateral upper and lower limbs and difficulty walking. MRI brain remained normal. MRI spine revealed lesions from C2-C4 and T1-T4 which progressed from previous scans). She received three days of intravenous methylprednisolone (1g/day) with minimal improvement. Two months later, she had an increase in her sensory symptoms, and was retreated with intravenous methylprednisolone (1g/day) and received two courses of rituximab. A further two months later she had a further deterioration with increasing paraesthesia and weakness in bilateral lower limbs. She was started on oral prednisone 40 mg po od with a slow taper planned as well as mycophenolate mofetil 1g bd. Progress imaging demonstrated hyperintensity and oedema from C2-C5, and C7-T7 with faint enhancement. She was retreated with rituximab six months later. A delay in rituximab retreatment with B cell repopulation as well as a reduction in weaning prednisone to 12.5 mg od heralded a further relapse 7 months later. She remains on maintenance prednisone, mycophenolate, and rituximab with B cell monitoring.

Diagnosis: MOG Ab-associated relapsing TM (short TM/LETM).

Adult 6

This 33 year old previously well Indian female presented with 10 days of right-sided eye pain with eye movement and visual acuity loss following a recent upper respiratory tract infection. MRI brain showed T2 hyperintensity and enhancement of the right optic nerve with no brain lesions. She was treated with 50 mg od of oral prednisone weaned over three weeks. Two weeks after steroid cessation, she represented with left eye pain on movement and visual loss. MRI demonstrated acute left optic neuritis with swelling, hyperintensity, and longitudinal enhancement; with persisting radiological changes of the right optic nerve. There were no brain lesions. MRI spine revealed T11 hyperintensity with no clinical correlate. She was once again started on 50 mg od of oral prednisone, once again with a 3 week planned taper. Fifteen months after this, she had a third episode of right-sided optic neuritis which was treated with oral prednisone 50 mg od weaned over ten days.

Diagnosis: MOG Ab-associated relapsing UON.
